# Deletion of *cox7c* Results in Pan-Azole Resistance in Aspergillus fumigatus

**DOI:** 10.1128/aac.00151-22

**Published:** 2022-06-01

**Authors:** Mingcong Chen, Guowei Zhong, Sha Wang, Peiying Chen, Lei Li

**Affiliations:** a Center for Global Health, School of Public Health, Nanjing Medical University, Nanjing, China; b Key Laboratory of Vector Biology and Pathogen Control of Zhejiang Province, Huzhou University, Huzhou Central Hospital, Huzhou, China; c School of Medicine and Life Science, Nanjing University of Chinese Medicine, Nanjing, China

**Keywords:** *Aspergillus fumigatus*, antifungal resistance, cytochrome *c* oxidase, reactive oxygen species, heme

## Abstract

In Aspergillus fumigatus, the most prevalent resistance to azoles results from mutational modifications of the azole target protein Cyp51A, but there are non-*cyp51A* mutants resistant to azoles, and the mechanisms underlying the resistance of these strains remain to be explored. Here, we identified a novel cytochrome *c* oxidase, *cox7c* (W56*), nonsense mutation in the laboratory and found that it caused reduced colony growth and resistance to multiantifungal agents. Meanwhile, we revealed that cold storage is responsible for increased tolerance of conidia to itraconazole (ITC) stress, which further advances azole-resistant mutations (cryopreservation→ITC tolerance→azole resistance). The deletion or mutation of *cox7c* results explicitly in resistance to antifungal-targeting enzymes, including triazoles, polyenes, and allylamines, required for ergosterol synthesis, or resistance to fungal ergosterol. A high-performance liquid chromatography (HPLC) assay showed that the *cox7c* knockout strain decreased intracellular itraconazole concentration. In addition, the lack of Cox7c resulted in the accumulation of intracellular heme B. We validated that an endogenous increase in, or the exogenous addition of, heme B was capable of eliciting azole resistance, which was in good accordance with the phenotypic resistance analysis of *cox7c* mutants. Furthermore, RNA sequencing verified the elevated transcriptional expression levels of multidrug transport genes. Additionally, lower itraconazole-induced reactive oxygen species generation in mycelia of a *cox7c*-deletion strain suggested that this reduction may, in part, contribute to drug resistance. These findings increase our understanding of how A. fumigatus’s direct responses to azoles promote fungal survival in the environment and address genetic mutations that arise from patients or environments.

## INTRODUCTION

Aspergillus fumigatus is a well-known and ubiquitous opportunistic fungal pathogen that plays an integral part in degrading organic biomaterials for carbon and nitrogen recycling in nature (1). It causes invasive or chronic infections that lead to a certain mortality rate every year, particularly for immunocompromised individuals, which are a challenge in global public health (1). At present, the treatment of invasive aspergillosis (IA) predominantly relies on triazoles, the first line of antifungal drugs (2). Members of the largest family of triazole agents primarily include fluconazole, itraconazole (ITC), posaconazole (PSC), and voriconazole (VRC). The latter is currently the first choice for the treatment of IA (3, 4). These commonly used systemic fungicides have free azole nitrogen, which binds to the iron atom of the heme, inhibiting the synthesis of ergosterol by 14α-lanosterol demethylase (Cyp51/Erg11) in the fungal cell membrane, and the inhibition of ERG5 has also been suggested (5, 6). However, the extensive use of antimicrobials over the decades has favored the development of resistance, and the resulting infections are often associated with a higher probability of antifungal treatment failure (7, 8). Moreover, the number of IA-specific antifungal drugs is limited; therefore, resistance to azoles is a growing problem for IA treatment and patient management (7, 8). Consequently, a comprehensive understanding of the molecular mechanisms underlying azole resistance may provide an avenue for therapeutic intervention against resistant strains.

To survive and thrive in harsh habitats, A. fumigatus has evolved effective response mechanisms, including smaller spores with rapid germination and dispersion capacities, phenotypic plasticity, and genetic alterations (9–11). Accordingly, several azole resistance mechanisms have been identified. The most common mechanisms of resistance in A. fumigatus are modifications of the *cyp51A* gene (12). Other vital mechanisms of azole resistance are mediated by the overexpression of multidrug resistance (MDR) pumps, metabolic adaptions, biofilm formation, and stress-response pathways (11, 13–15). There are two categories of *cyp51A* modifications, tandem insertions at the *cyp51A* promoter and more than 20 coding region point mutations (e.g., TR34/L98H, G54W, P216L, M220V/K/T, and G448S). These modifications cause the overexpression of Cyp51A or reduce its binding affinity to azoles (11, 16), which is associated with the acquisition and development of A. fumigatus resistance to azole activity. Additionally, mutation or overexpression in *cyp51B* also results in triazole resistance (17).

Although such *cyp51A*-based azole-resistant strains have been reported frequently in the past years, variants without any mutation in the *cyp51A* gene that show low susceptibilities to azole drugs have emerged. Such *cyp51A*-free mechanisms, which were first identified in clinical cases, are thought to be responsible for treatment failure (4). Recently, studies investigating mutations acquired in laboratory strains of triazole-susceptible A. fumigatus that develop resistance upon triazole exposure have identified potentially novel resistance mechanisms (11, 18). Thus, so far, no less than 10 mutations other than in *cyp51* leading to azole resistance have been frequently identified. The majority of these mutations are located in genes involved in the regulation or biosynthesis of ergosterol, maintenance of mitochondrial respiration, and transporters (19–21). These studies significantly increase our understanding of the mechanisms underlying resistance to antifungal drugs, and the non-Cyp51A azole-resistant mutations are becoming the focus of attention.

Mitochondrial dysfunction affects azole resistance through potential mechanisms in fungi, for example, regulation of drug efflux pumps, iron homeostasis (22), decreased reactive oxygen species (ROS) production (23), and activity of cytochrome *c* oxidase (Cox/Cco; also known as complex IV) (24). Cytochrome *c* oxidase, the terminal component of the mitochondrial respiratory chain, catalyzes the electron transfer from reduced cytochrome *c* to oxygen. Cox is a heteromeric complex, and the number of subunits varies from 3 to 5 in bacteria and up to 12 and 13 in yeast and mammals, respectively. It is encoded by mitochondrial genes (Cox1p, -2p, and -3p, which function in electron transfer) and nuclear genes (which function in the regulation and assembly of the complex) (25). Mitochondrial fission mutants Δ*dnm1* and Δ*fis1* showed decreased activity of complex IV and increased VRC resistance in A. fumigatus (24). The amino acid substitution R243Q in the *cox10* gene of A. fumigatus was induced in the laboratory and reported to cause azole resistance (21, 26). In addition, amino acid substitutions P17S and A423V are present in the *cox10* gene of A. fumigatus strains originating from clinical and environmental sources; however, none of these substitutions have been validated to confer azole resistance. Cox10 is a farnesyltransferase that plays a critical role in the mitochondrial heme biosynthetic pathway by catalyzing the conversion of heme B to heme O, followed by Cox15p conversion to heme A, which presents a cofactor required for the stability and folding of the Cox1 subunit. Cox10- and Cox15-null mutants exhibit identical phenotypes, having colony growth defects and multidrug resistance (21). Deleting the *Afcox10* gene results in the activation of Ca^2+^ signaling and calcineurin-dependent response element drug transporters, which results in azole-resistant phenotypes.

In a previous ITC susceptibility assay, we used the A. fumigatus A1160^C^ spore suspension, which had been kept in sterile deionized water at 4°C for several weeks as a wild-type control; unexpectedly, two independent azole-resistant mutants, IM1 and IM2, arose spontaneously from the ITC-containing (0.2 μg/mL) YAG plate. This interesting discovery intrigued us to identify the potential genome mutations that induce this drug-resistant phenotype. Therefore, through next-generation sequencing analysis, a *cox7c* (W56*) mutation not reported before was isolated from both mutants. We further determined experimentally that *cox7c* is involved in multiple antifungal resistance *in vitro*. More importantly, we provided evidence that the lack of *cox7c* influences the heme B content, the regulation of a series of metal-ion metabolism-related genes and drug efflux genes, and ROS generation. Thus, the novel *cox7c* mutation may help A. fumigatus survive azole exposure and confer azole resistance.

## RESULTS

### Identification of the induced *cox7c* mutation involved in azole resistance.

**FIG 1:**
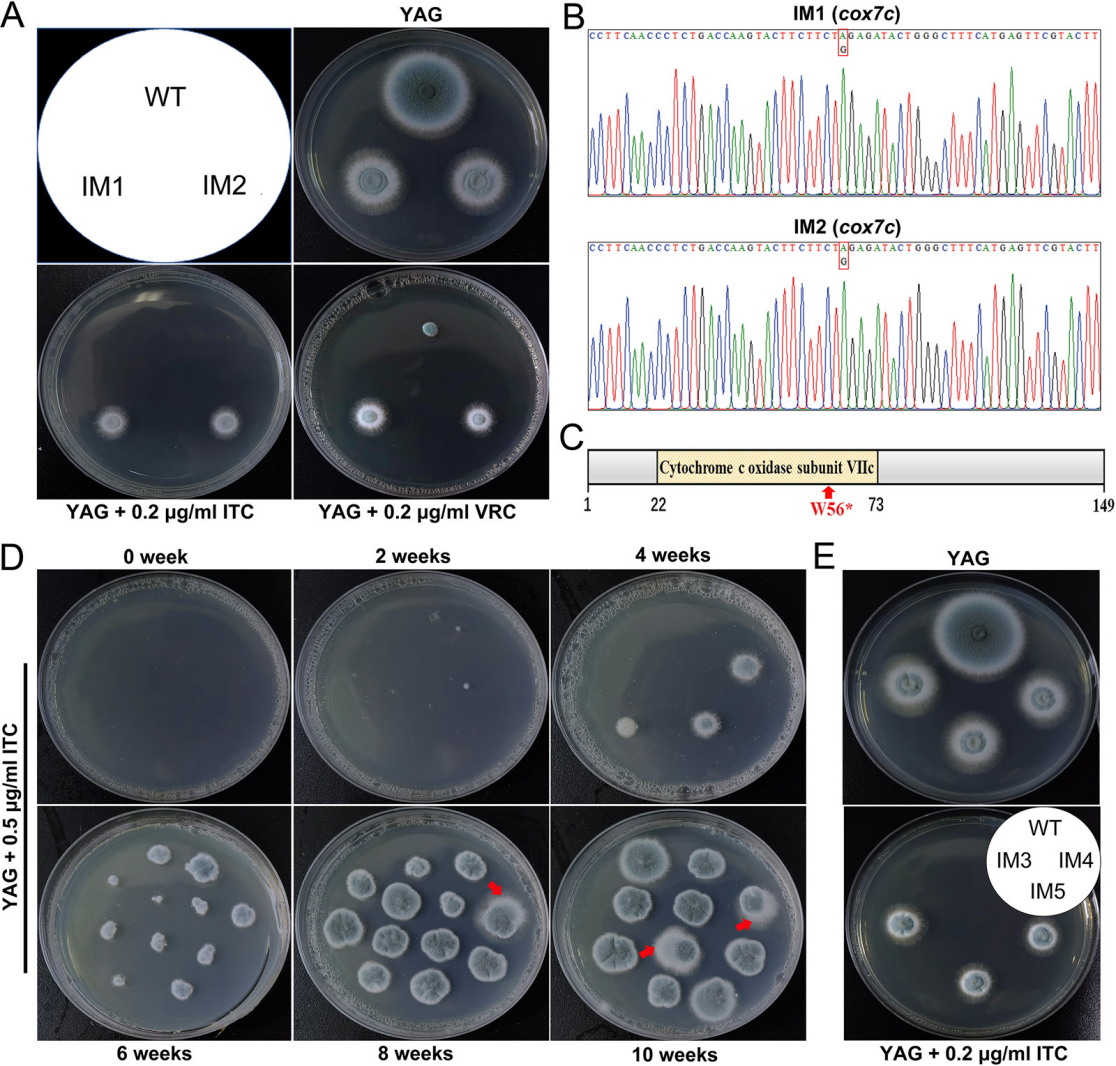
Two A. fumigatus isolates resistant to azoles have the *cox7c* (W56*) nonsense mutation. (A) Comparison of the susceptibility of the reference strain A1160^C^, IM1 mutant, and IM2 mutant to ITC or VRC. Strains were grown on YAG agar plates supplemented with or without ITC or VRC at the indicated concentrations and incubated at 37°C for 2 days. (B) Analysis of *cox7c* sequencing map of IM1 and IM2 mutants. (C) Schematic diagram of the conserved domain and the identified mutation site of Cox7c. (D) Wild-type spores exposed to different cryopreservation times were cultured on YAG agar plates supplemented with 0.5 μg/mL ITC at 37°C for 4 days. Tested spores were cryopreserved in sterile deionized water at −20°C for 0 to 10 weeks. Red arrows indicate the breakthrough growth of ITC-more tolerant mutants IM3, −4, and −5. (E) Selected mutated clones were purified and subcultured on YAG agar plates supplemented with or without ITC at 37°C for 2 days.

Here, the two ITC-resistant isolates we recently serendipitously discovered were named ITC-induced mutant (IM) 1 and 2. As shown in Fig. 1A, on YAG plates, these two mutants exhibited similar defects in colony phenotype to the starting strain A1160^C^, suggesting that the mutated genes were required for normal vegetative growth. However, IM1 and IM2 showed low susceptibility levels to ITC and VRC compared with the A1160^C^ strains, indicating the existence of acquired azole-antifungal resistance mechanisms. To verify the mutation sites, we surveyed the literature and cloned coding regions of the *cyp51A*, *erg3*, *erg5*, *erg6*, *srbA*, *hapX*, *hapB*, *hapC*, *hapE*, *hmg1A*, *hmg1B*, and *cox10* genes, including their promoter regions, from IM1 and IM2. However, the sequencing results indicated that no mutations occurred in these selected genes. To unravel the potential mutations responsible for azole resistance, next-generation sequencing was performed on IM1, IM2, and the wild-type strain (A1160^C^). After a comparative genomic analysis, approximately 490 single nucleotide polymorphisms (SNPs) were detected in the coding regions of both IM1 and IM2 (see Data Set S1 in the supplemental material). Because IM1 and IM2 showed almost the same phenotype and were both derived from A1160^C^, we hypothesized that they contained a consensus mutation site distinct from A1160^C^. Finally, we identified a unique SNP embedded in the predicted protein AFUB_064240 that harbored an amino acid change from tryptophan (TGG, W) to stop (TAG) at codon 56 (W56*), which was confirmed through single-gene sequencing (Fig. 1B).

The Conserved Domain Database (CDD) revealed that full-length AFUB_064240 contains 149 amino acid residues and has one predicted domain cytochrome *c* oxidase subunit VIIc starting and ending at positions 22 and 73, respectively (Fig. 1C). Thereafter, we designated the AFUB_064240 gene *cox7c* and referred to the corresponding protein as Cox7c. A BLAST search of the NCBI database revealed that Cox7c homologs are widely present in fungi and metazoans but are absent in plants. It shares 21.3% and 33.3% sequence identities with Saccharomyces cerevisiae and Schizosaccharomyces pombe Cox8p, respectively. The phylogenetic relationships among these homologs for selected organisms were analyzed, and a phylogenetic tree was constructed (Fig. S1). Cox7c homologs form two clusters, fungal and metazoan. Among these proteins, A. fumigatus Cox7c had the highest identity score (82.7%) with its homolog in Aspergillus nidulans and the lowest identity score with its homolog Cox8p from S. cerevisiae (21.3%). These results demonstrated the conservation of the Cox7c amino acid sequence among these organisms.

### Long-term cryopreservation of spores tends to produce ITC-induced mutagenesis.

To investigate the causes of such *cox7c* mutation, we conducted cryopreservation time series and azole induction analyses on the A. fumigatus A1160^C^ and Af293 strains during incubation with ITC to see if we could recover the *cox7c* mutation. While ITC tolerance was seen (Fig. S2A and S3) and, subsequently, three more ITC-tolerant mutants were further identified (IM3, -4, and -5) (Fig. 1D and E), none of these harbored the *cox7c* mutation. The ITC (MIC, 2 μg/mL) and VRC (MIC, 1 μg/mL) MICs were also raised uniformly for these three mutants. Additionally, the azole tolerance/resistance of long-term cryopreserved spores only occurs against ITC among these antifungals (Fig. S2). These results suggest that cold storage does not directly select for a mutation that provides cross-resistance to azoles but just increases ITC tolerance, allowing for the selection of azole-resistant mutations under subsequent specific ITC stress (cryopreservation of conidia first and ITC exposure later, that is, cryopreservation→ITC tolerance→azole resistance).

### Deletion of *cox7c* results in different levels of antifungal susceptibility.

**FIG 2:**
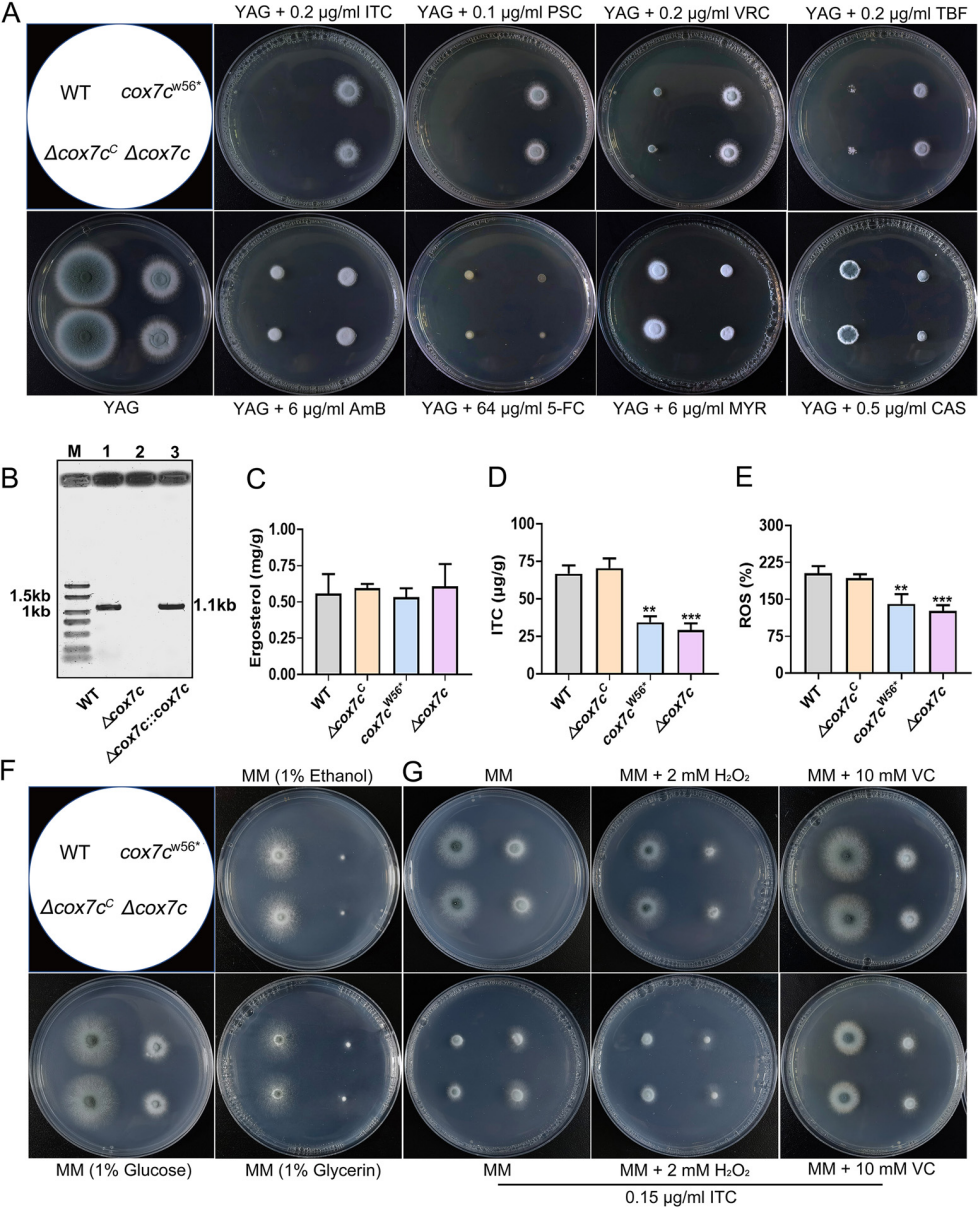
Deletion or mutation of *cox7c* results in lower intracellular ITC retention, decreased ROS, and different-level resistance of A. fumigatus to antifungal drugs. (A) Cross-resistance and sensitivity tests to ITC, VRC, PSC, TBF, AmB, MYR, 5-FC, and CAS in selected strains. All strains were grown on indicated plates at 37°C for 2 days. (B) Diagnostic PCR using genomes as templates confirmed that the full-length sequence of *cox7c* was replaced by the selective gene in the deletion strain and was complemented in the reconstitution strain. For lanes 1, 2, and 3, the PCR primer pair was *cox7c* P2/Diag *cox7c* to detect whether there was a *cox7c* gene, and the expected size was 1.1 kb. (C) Comparison of the intracellular ergosterol concentrations in the displayed strains. (D) Intracellular ITC concentrations in strains stimulated by 1 μg/mL ITC for 2 h. The concentration values of ITC and ergosterol were analyzed by one-way analysis of variance (ANOVA) with unpaired Student's *t* test and presented as the means ± SD of three biological samples. Error bars represent the corresponding standard deviations. **, *P < *0.01; ***, *P < *0.001. (E) Increment of ITC-induced ROS in mycelia of indicated strains. Fluorescence intensity values were presented as the means ± SD of three biological replicates and analyzed by one-way ANOVA with unpaired Student's *t* test (**, *P < *0.01; ***, *P < *0.001). (F) Colony phenotypes of indicated strains on MM containing glucose, ethanol, or glycerol, respectively, as a sole carbon source at 37°C for 2 days. (G) Colony phenotypes of indicated strains on MM containing H_2_O_2_ or VC in the presence or absence of ITC at 37°C for 2 days.

To further confirm that the mutated *cox7c* gene caused the defective growth and azole resistance phenotypes of the IM1 and IM2 mutants and provide insight into its function, the entire coding region of *cox7c* was deleted to generate the Δ*cox7c* mutant. The knockout strain was constructed by gene deletion in the A1160^C^ background strain and was subjected to diagnostic PCR analysis. As shown in Fig. 2B, in the Δ*cox7c* mutant, *cox7c* was successfully replaced by the selective Neurospora crassa *pyr4* marker gene. In addition, we reconstituted the complementation strain Δ*cox7c*^C^ by using the full-length *cox7c* gene cassette. Compared with wild type and Δ*cox7c*^C^, the Δ*cox7c* mutant showed reduced colony size and ITC or VRC resistance, similar to the IM1 mutant (Fig. 2A).

To further determine whether the absence of *cox7c* affects cross-resistance to antifungals, we selected several antifungals with different action mechanisms to assess responses to azoles (posaconazole [PSC]), polyenes (amphotericin B [AmB]), allylamines (terbinafine [TBF]), nucleoside analogs (5-fluorocytosine [5-FC]), sphingolipid synthesis inhibitors (myriocin [MYR]), and echinocandins (caspofungin [CAS]). Notably, a set of drug-tolerant phenotypes was classified into three categories according to the diversity of colony size on plates when compared to the control strain, A1160^C^ (Fig. 2A). In class I, the *cox7c* mutants showed the highest degree of resistance to ITC, PSC, and VRC compared with the wild type when spotted on solid YAG media containing tested antifungals. In class II, the *cox7c* mutants were cross-tolerant to AmB and TBF. However, in classes III, all the mutants and the wild-type strain displayed 5-FC-, MYR-, and CAS-susceptible phenotypes. Subsequently, we tested the MICs of the *cox7c* mutants and the wild-type strain (A1160^C^) in response to antifungals following the Clinical and Laboratory Standards Institute M38-A2 standard methods (27), and the results are provided in Table 1. In agreement with the plate tests, the MIC assays further confirmed that the *cox7c* mutants exhibited increased tolerance levels to azoles AmB and TBF. The azole MICs of *cox7c* mutants were 4-fold (4 μg/mL in Δ*cox7c* versus 1 μg/mL in wild type) higher, and the AmB and TBF MICs of Δ*cox7c* were 2-fold higher than that of A1160^C^. In contrast, there were no detectable differences in the MICs of MYR and 5-FC among these strains. In summary, *cox7c* mutants have a specific antifungal resistance profile and exclusively antagonize antifungals inhibiting ergosterol biosynthesis or targeting fungal ergosterols, suggesting the existence of a potential novel antifungal resistance mechanism.

**TABLE 1:** MICs of ITC, VRC, PSC, AmB, TBF, MYR, and 5-FC for selected isolates^*a*^

Strain	MIC (μg/mL) of:						
	ITC	VRC	PSC	AmB	TBF	MYR	5-FC
A1160^c^	1	0.5	0.5	1	4	32	>128
*cox7c*^W56^*	4	2	2	2	8	32	>128
Δ*cox7c*	4	2	2	2	8	32	>128

*^a^*ITC, itraconazole; VRC, voriconazole; PSC, posaconazole; AmB, amphotericin B; TBF, terbinafine; MYR, myriocin; 5-FC, 5-fluorocytosine.

It has been suggested that azoles interfere with the activity of 14-α-lanosterol demethylase to inhibit ergosterol biosynthesis, and the ergosterol level is a determinant for resistance to any class of azoles (6). To interpret the resistance mechanism, we first tested and compared the ergosterol contents between the *cox7c* mutants and the A1160^C^ strain. Total sterol was extracted and detected by high-performance liquid chromatography (HPLC) analysis. However, no significant differences in the ergosterol contents were found among these strains (Fig. 2C). Furthermore, the intracellular ITC contents of the *cox7c*-null mutant and the wild-type strain were directly measured by HPLC, and the intracellular ITC retention in the mutants, expectably, was 2.3-fold lower than that in the reference strain (Fig. 2D). We reasoned that the reduced ROS content may alleviate the fungicidal effects of antifungals in mutants, and indeed, we observed less intracellular ROS accumulation in the mutants (only 63% that of the wild type) when the most representative azole ITC was used as an ROS stimulator (Fig. 2E). Less production of ROS was also detected in the mutants with or without H_2_O_2_ stimulus in comparison to the wild type (Fig. S4A). As shown in Fig. 2F, *cox7c*^W56^* and Δ*cox7c* showed nonfermentable carbon source colony defects in minimal medium (MM) plates supplemented by glycerin or ethanol as the sole carbon source compared with the robust hyphal growth of the parental wild-type and complemental strains, suggesting that *cox7c* mutation could cause loss of mitochondria aerobic respiration capacity, which may account for the decreased ROS in mutants. Moreover, when H_2_O_2_ was added to the media in the presence or absence of ITC, *cox7c* mutants exhibited a slower growth rate and decreased resistance to ITC compared with parental wild-type and complemental strains (Fig. 2G). Interestingly, the antioxidant reagent vitamin C (VC) improved the wild-type strain’s ITC tolerance but not the mutants’ (Fig. 2G). Taken together, these results suggest that the intracellular ITC and ROS decreases in the *cox7c* mutants are responsible for their azole resistance. However, more in-depth studies are needed to fully explain the mechanisms of *cox7c* depletion-mediated azole adaptation of A. fumigatus.

### Δ*cox7c* differentially expressed genes are primarily involved in transporter activity and iron metabolism.

**FIG 3:**
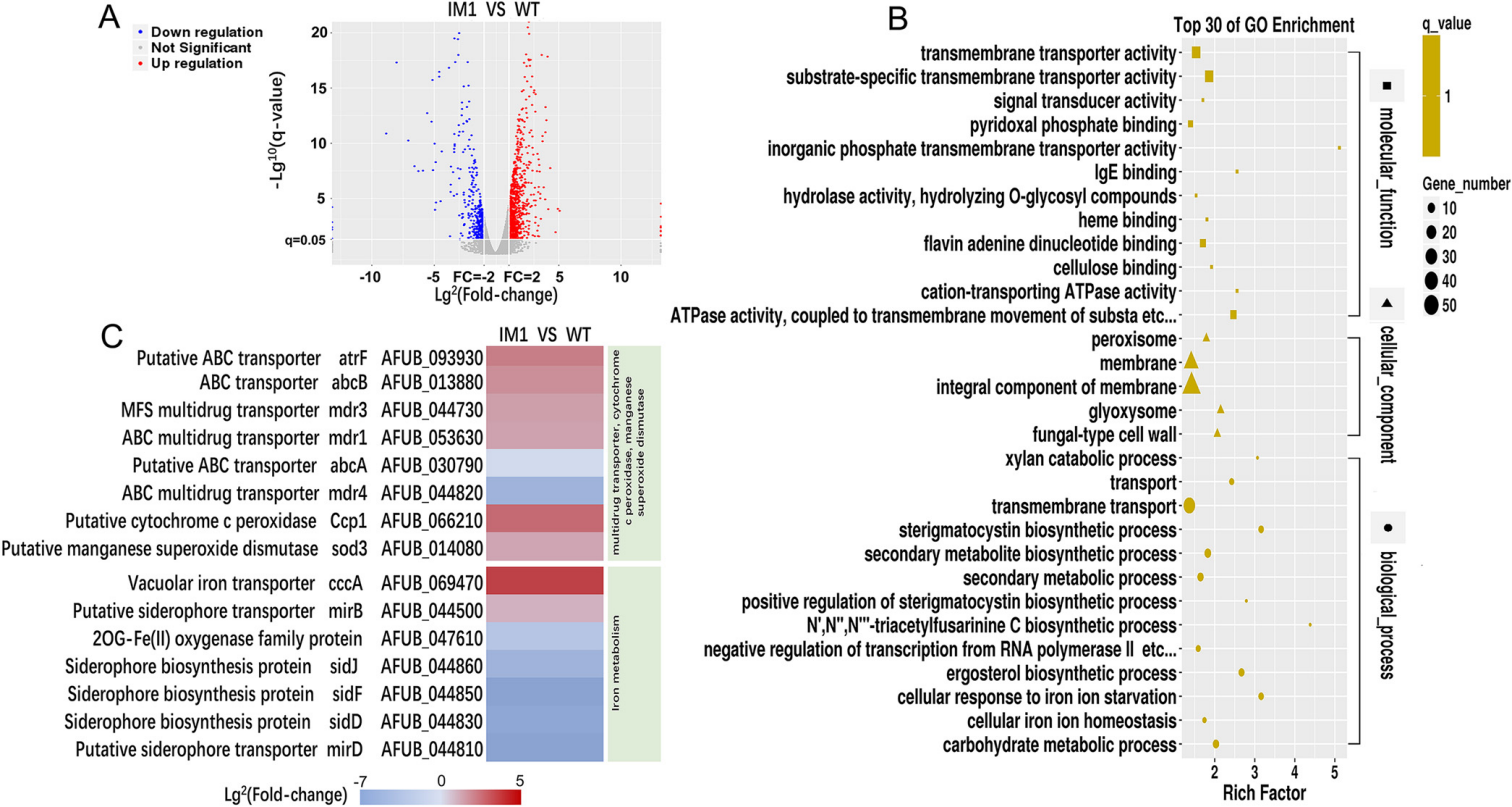
The IM1 mutant has significant changes in the patterns of gene transcription. (A) Volcano plot highlighting downregulated and upregulated genes with a |(lg_2_FC)| of ≥1 and a *P* value of ≤0.05. Differential gene expression in the IM1 mutant compared with the parental wild type was measured by RNA sequencing. Red, upregulation; blue, downregulation; gray, not significant. (B) GO term enrichment analysis of RNA-seq data. Rich factor, the ratio of input DEGs that are annotated in a term to all genes that are annotated in this term. (C) Heatmap analysis of RNA-seq results for the annotated genes encoding proteins putatively involved in multidrug transport, ROS scavenging, and iron metabolism. Log_2_ fold changes are plotted for significant difference.

A transcriptome sequencing (RNA-seq)-based approach was used to further explore the *cox7c* mutation-incurred antifungal-resistant mechanisms. Two groups of RNA samples from the IM1 mutant and the wild-type strain A1160^C^ (serving as the control) were prepared independently for transcriptome analysis. The comparative RNA-seq analysis revealed that the gene expression profile of the IM1 strain was greatly different from that of the A1160^C^ strain. Among the 10,041 A. fumigatus transcripts, 1,306 were significantly regulated (*P ≤ *0.05, −1 ≥ log_2_ fold change [log_2_ fold change] ≥ 1) (Data Set S2). Of the 1,306 differentially expressed genes (DEGs) between the two experimental groups, 890 were upregulated, and 406 were downregulated (Fig. 3A and Data Set S2). As shown by the gene ontology (GO) functional enrichment analysis, the DEGs were primarily involved in the transporter activity, cellular response to iron ion starvation, cellular iron ion homeostasis, etc. (Fig. 3B).

In addition, a heatmap showing the selected top upregulated or top downregulated DEGs involved in multidrug transporter and iron metabolism was constructed (Fig. 3C). Most genes involved in the multidrug transporter from the IM1 group, in contrast to the control group, were upregulated, including *atrF* (fold change, 4.37), *abcB* (FC, 3.34), *mdr3* (FC, 2.65), and *mdr1* (FC, 2.55). However, *abcA* (FC, 0.72) and *mdr4* (FC, 0.03) were downregulated. Additionally, putative cytochrome *c* peroxidase *ccp1* and putative manganese superoxide dismutase *sod3* genes increased by 5.92- and 2.41-fold. Among the metal metabolism-related genes, iron metabolic genes showed the most striking alteration in terms of number and fold change. For instance, the transcription level of the vacuolar iron transporter *cccA* (AFUB_069470) showed a dramatic 11.4-fold upregulation. Simultaneously, the transcription levels of siderophore biosynthesis *sidJ* (AFUB_044860), *sidF* (AFUB_044850), *sidD* (AFUB_044830), and siderophore transporter *mirD* (AFUB_044810), as well as 2OG-Fe (II) oxygenase family genes (AFUB_047610), were 29.38-, 462.13-, 95.84-, 259.47-, and 8.17-fold less, respectively, than those of the wild-type strain. The transcriptional expression of predicted metallothionein (AFUB_098700) was remarkably enhanced by 31.60-fold. These changes in gene expression may represent adaptations of the *cox7c*-null mutant to a cytoplasmic iron excess condition. Indeed, our results showed the total intracellular iron concentration of Δ*cox7c* was significantly higher than that of the wild-type strain (Fig. S4B). In addition, genes involved in other divalent cations’ homeostasis, such as zinc, manganese, and magnesium, were not obviously regulated. Unlike in a previous study (21) in which Ca^2+^ signaling mediated Δ*cox10*-related azole resistance, mRNA levels of Ca^2+^-signaling calmodulin and transcription factor *crzA* in the *cox7c* mutant remained unchanged statistically. Additionally, *cyp51A* and *cyp51B* expression increased by 1.79- and 1.4-fold, respectively, but they were not statistically regulated.

### *cox7c* may increase azole resistance by regulating metal ions.

**FIG 4:**
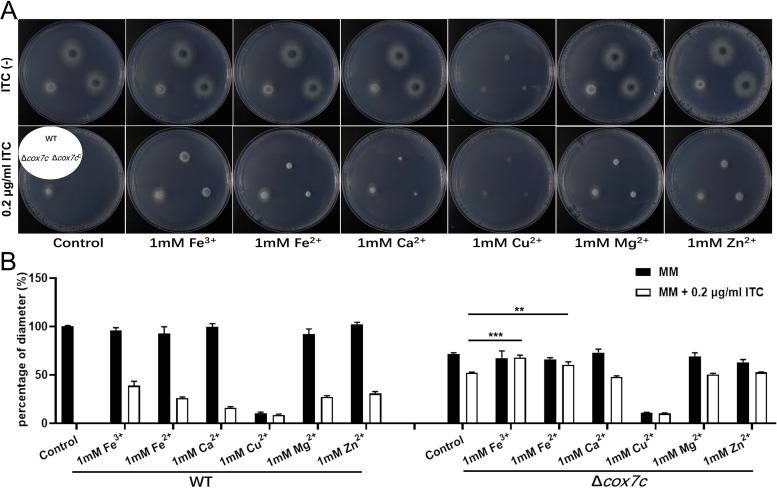
Addition of metal ions changes the azole susceptibility of A. fumigatus. (A) Comparison of the susceptibility of the indicated strains to 0.2 μg/mL of ITC, with the addition of various kinds of metal ions. All strains were grown on MM plates at 37°C for 2 days. (B) Colony sizes, shown as percentages of the diameter of the control strain (A1160^C^), for the indicated strains cultured on MM plates in the presence of 1 mM metal ions and with or without 0.2 μg/mL of ITC. Values are means and SD from three independent colonies (**, *P < *0.01; ***, *P < *0.001 [unpaired Student's *t* test]).

**FIG 5:**
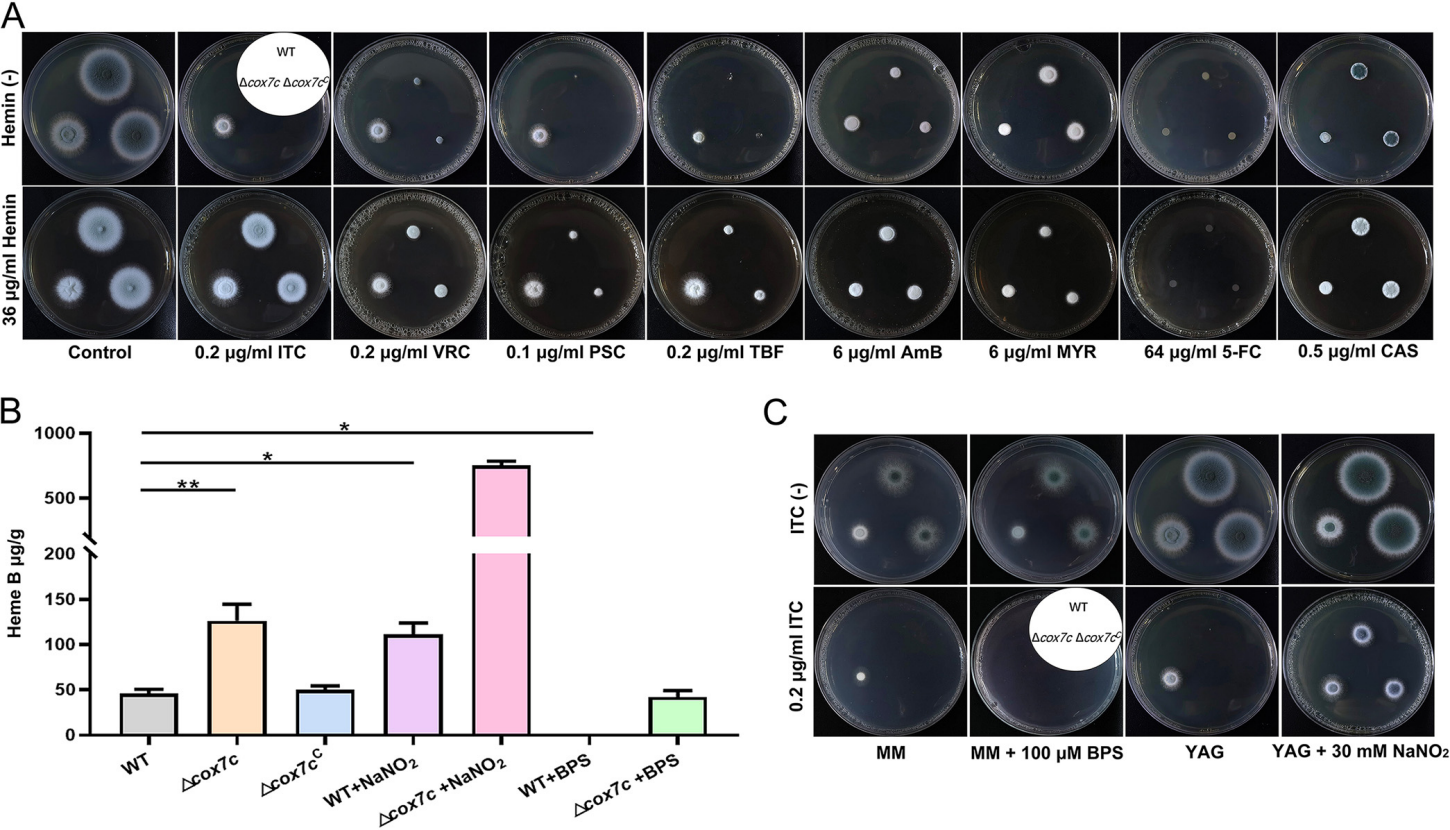
Heme promotes the azole resistance of A. fumigatus. (A) Comparison of colonies of A1160^C^, Δ*cox7c*, and Δ*cox7c*^C^ strains on YAG plates in the absence or presence of the indicated concentrations of antifungals (ITC, VRC, PSC, TBF, AmB, MYR, 5-FC, and CAS), with the addition of 36 μg/mL hemin. Strains were grown on indicated plates at 37°C for 2 days. (B) HPLC analyses of the intracellular concentrations of heme in the displayed strains. The hemin concentration values were analyzed by one-way ANOVA with unpaired Student's *t* test and presented as the means ± SD of three biological samples. Error bars represent the corresponding standard deviations. *, *P < *0.05; **, *P < *0.01. (C) Comparison of the susceptibility of A1160^C^, Δ*cox7c*, and Δ*cox7c*^C^ to ITC, with the addition of 100 μM BPS or 30 mM NaNO_2_. Strains were grown on indicated plates at 37°C for 2 days.

On the basis of the transcriptomic data, we sought to determine whether metal homeostasis changes induced by supplementation with metal ions could change A. fumigatus’s susceptibility to azoles or if they were responsible for the azole resistance mechanism of Δ*cox7c.* Here, we compared the growth phenotypes of Δ*cox7c* and its parental strain when supplemented with or without metal ions (FeCl_3_, FeCl_2_, CuSO_4_, MgCl_2_, ZnCl_2_, and CaCl_2_) and ITC. As shown in Fig. 4A and B, the addition of 1 mM metal ions into the MM plates without ITC produced no statistical difference between the colony growth of wild type and Δ*cox7c.* In contrast, when cultured in media supplemented with 0.2 μg/mL ITC and 1 mM various respective metal ions, the wild-type strain displayed enhanced tolerance to the ITC. Notably, in the presence of 0.2 μg/mL of ITC, the addition of iron ions increased the growth rate of both the wild-type and the *cox7c* deletion strains. To further assess whether the iron status was involved in ITC resistance, the Δ*cox7c* mutant was exposed to the iron starvation medium supplemented with 100 mM of the iron-specific chelator bathophenanthroline disulfonic acid (disodium salt) (BPS). As predicted, the ITC-sensitive morphology was restored in Δ*cox7c* (Fig. 5C). Therefore, we concluded that metal ion metabolism contributed to the ITC resistance of A. fumigatus, and there may be a link between iron metabolism and *cox7c*.

### Intracellular heme B mediates susceptibility to ITC of Δ*cox7*.

Based on the differential expression analysis of transcriptomic data, cellular iron ion homeostasis-associated genes were investigated. Heme, an iron porphyrin compound, is the auxiliary group of hemoglobin, myoglobin, cytochrome, peroxidase, nitric oxide synthase/nitrite reductase/nitric oxide reductase, and catalase (28, 29). Considering heme’s affinity for iron ions and the influence of iron ions on the adaptation of A. fumigatus to ITC, we hypothesized that the *cox7c*-related azole resistance might be associated with intracellular heme. We performed an antifungal resistance evaluation using the addition of hemin (an oxidized or *in vitro* form of heme). The external addition of 36 μg/mL of hemin distinctly caused the wild-type and the reconstituted strains to be resistant to antifungal drugs targeting enzymes in the ergosterol biosynthesis pathway or fungal ergosterols (Fig. 5A), although it did not remarkably alter the resistance of the *cox7c* mutants (except for ITC and TBF). This phenomenon was not observed for the other three drugs (MYR, 5-FC, and CAS), which corroborated our previous observations (Fig. 2A). Thereafter, to prove that the *cox7c*-mediated antifungal adaptation specifically involved heme, an HPLC analysis of heme was performed. A comparison with the structure of the standard substance revealed that Δ*cox7c* had markedly more intracellular heme B than A1160^C^ (Fig. 5B), which suggested that *cox7c* may play a role in the conversion of heme B to heme derivatives (30). To test if other ITC-resistant mutants also have an increase in the intracellular heme B concentration, we further selected two azole-resistant mutants, Δ*Afssn3* and 1160-331(*cyp51A^G54W^*), for heme B analysis. As shown in Fig. S4C, no significant increase was found in the intracellular heme B content of these two mutants. In addition, the heme B content in the wild-type strain rose significantly in the presence of ITC but not in the presence of AmB, TBF, or CAS (Fig. S4D). Therefore, the resistance to specific antifungal agents caused by the deletion of *cox7c* and elevated intracellular heme B concentrations were strongly correlated.

It is widely believed that NaNO_2_ causes nitric oxide (reactive nitrogen species) damage in cells (31), and intracellular heme interacts with nitric oxide during its metabolism (32, 33). To further verify heme’s performance in azole resistance, NaNO_2_ was used to alter the heme concentration in A. fumigatus cells. When sodium nitrite was added to YAG solid medium containing 0.2 μg/mL of ITC, the wild-type strain produced breakthrough growth (Fig. 5C), which had an ITC resistance near the level of Δ*cox7c*. An HPLC analysis also demonstrated that a higher heme B content was produced in the wild-type cells following sodium nitrite stimulation (Fig. 5B). Under BPS chelation conditions, in which the resistance of Δ*cox7c* declines, the heme B concentration dropped dramatically both in wild type and Δ*cox7c* (Fig. 5B and C).

**FIG 6:**
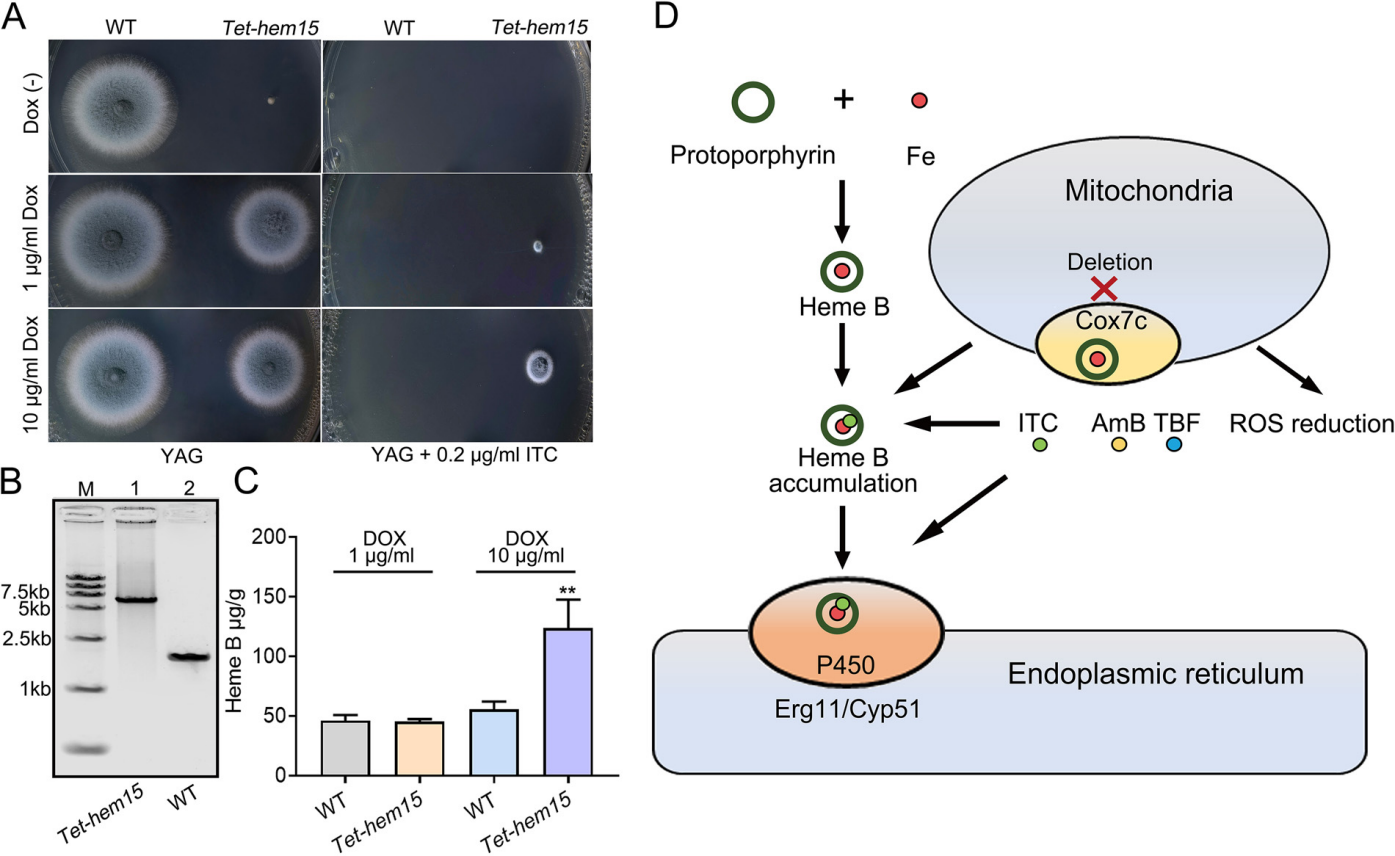
Overexpression of Hem15 in heme biosynthesis pathway affect azole resistance. (A) Comparison of colonies of parental wild-type A1160^C^ and *Tet-hem15* strains on YAG plates in the absence or presence of the indicated concentrations of doxycycline (Dox) or ITC at 37°C for 48 h. (B) PCR analysis (amplified using primer pair tet-hem15 P1/P6) revealed successful construction of the *Tet-hem15* mutant. For lanes 1 and 2, PCR product electrophoresis showed the expected 6,245-bp and 1,867-bp bands, respectively. (C) HPLC analyses of the intracellular concentrations of heme B in the displayed strains. The heme concentration values were analyzed by one-way ANOVA with unpaired Student's *t* test and presented as the means ± SD of three biological samples. Error bars represent the corresponding standard deviations. **, *P < *0.01. (D) Schematic showing the *cox7c* depletion contributes to fungal drug resistance through inducing heme B accumulation and ROS reduction.

Furthermore, a *Tet-hem15* (AFUB_055280; a putative mitochondrial ferrochelatase catalyzes the last step of heme biosynthesis) (34) mutant was constructed in which the promoter of *hem15* was replaced with a conditional doxycycline (Dox)-inducible promoter (Fig. 6B). The *Tet-hem15* mutant was greatly inhibited when grown on a YAG plate without doxycycline (tet-off). Conversely, the severely impaired growth was almost complemented when 1 μg/mL Dox was added into the medium (tet-on) (Fig. 6A). The susceptibility of the *Tet-hem15* mutant to ITC was determined. As predicted, the *Tet-hem15* strain accumulated its heme B content and was resistant to ITC when it was cultured on YAG supplemented with Dox at the concentration of 10 μg/mL (overexpression) (Fig. 6A and C), and this resistance phenotype of *Tet-hem15* was almost restored to normal under the tet-on condition (Fig. 6A), suggesting *hem15* could regulate azole susceptibility. As a result, we verified the hypothesis that Δ*cox7c* resistance to azole is mediated by intracellular heme. We examined our transcriptomics data; however, there were no statistical differences among the heme biosynthetic genes at the mRNA level between the two groups.

## DISCUSSION

In this study, we identified a novel *cox7c* (W56*) mutation from two A1160^C^-derived ITC-resistant isolates (obtained after exposure of long-term-preserved A. fumigatus A1160^C^ spores to ITC) and characterized the function of Cox7c, a subunit of mitochondrial respiratory chain complex IV, in A. fumigatus. We showed that the loss of Cox7c is not essential for the mold’s viability but, indeed, lessens its vegetative growth and, interestingly, antifungal susceptibility. Interestingly, the azole resistance of most mitochondrial dysfunction mutants is at the cost of reduced growth rates. The *cox7c* (W56*) mutation results in a genetic trade-off in which A. fumigatus is harmed in the normal environment (reduction in the growth rate) but benefits under fungicide selection *in vitro* (multiple antifungal resistance).

The Cox7c subunit (Cox8p in yeast) is found only in eukaryotes, and little specific functional information is known. The lack of Cox8 in the genomes of green plants, as determined by bioinformatics analyses, is intriguing because it is crucial for cellular respiration and ATP production. Thus, there may be an evolutionally functional compensation to maintain its activity. In yeast, Cox8p-null mutants show a lower respiratory growth rate and decreased competitive fitness (35). However, none of the amino acid substitutions in *cox7c* have been previously reported as being involved in fungal azole resistance.

Cox7c is 149 amino acids in length (Fig. 1C), and it is predicted to possess a conserved domain with remarkable similarity to cytochrome *c* oxidase VIIc. Multiple alignments of Cox7c indicated it shares a low sequence identity within organisms, showcasing a divergence in the evolutionary process (see Fig. S1 in the supplemental material). On the basis of these bioinformatics analyses, we speculate that the Aspergillus Cox7c, in addition to oxidative phosphorylation, has unique biological functions compared to other fungi. Likewise, the particular VIIc subunit in A. fumigatus may have been assigned specific functions different from other subunits. Here, we demonstrated that an amino acid alternation (W56*) or full-length deletion of *cox7c* caused a decrease in intracellular azole concentration, mitigating the fatal effects of ergosterol deprivation. The question is, what components are involved in the reduction of intracellular azole retention?

The mitochondrial iron transporter MrsA coordinates iron homeostasis. The deletion of *mrsA* induces abnormal ROS accumulation and hypersusceptibility to azole or oxidative stresses (36). A. fumigatus SREBP SrbA mediates triazole response and regulation of iron acquisition in response to hypoxia and low-iron conditions. The exogenous addition of high iron partially rescues the triazole susceptibility and decrease in ergosterol content phenotypes of Δ*srbA* (37). Transcriptome-based iron metabolism-related gene expression changes are primarily involved in the cellular response to iron ion starvation and cellular iron ion homeostasis (Fig. 3B and C); therefore, it is likely that the *cox7c* mutant also disrupts mitochondrial or cytoplasmic iron homeostasis, incurring azole resistance. Null mutants of HapX, a bZIP transcription factor, have a reduced ability to produce siderophores, are attenuated in virulence, and increase tolerance to triazoles (19). These phenotypes somewhat resemble our findings for *cox7c* mutants. However, the mRNA level of *hapX* was not remarkably altered in *cox7c* mutants (fold change of 0.75), and the same results were obtained in the analysis of the CCAAT-binding complex subunits *hapB*, *hapC*, and *hapE*, suggesting a potential interaction between *cox7c* and the transcriptional regulation of genes involved in iron homeostasis independent of *hapX*. The addition of ferrous iron ions significantly increased the azole resistance level of A. fumigatus (Fig. 4A). Iron is the central atom of heme, an indispensable component of cytochrome (38). In this research, the loss of *cox7c* in A. fumigatus increased the accumulation of heme B, the most abundant of the hemes. This may result from mitochondrial dysfunction and iron metabolism-related disorder. The extracellular addition of hemin or intracellular accumulation of heme B elevated the resistance/tolerance of the wild-type strain primarily to azoles (ITC, VRC, and PSC) and then polyenes (TBF) and allylamines (AmB), whereas it did not affect resistance to other antifungals (Fig. 5A and B). The effects of extracellular hemin supplementation on antifungal susceptibility was consistent with that caused by the *cox7c* deletion. Additionally, the indirect inhibition of heme biosynthesis by BPS chelation and the promotion of heme accumulation by NaNO_2_ or by overexpression of *hem15* led to decreasing azole resistance or increasing azole resistance comparable to that of the *cox7c*-deletion mutant, respectively (Fig. 5 and 6). These results suggest that the *cox7c*-mediated antifungal drugs’ adaptation in A. fumigatus is achieved by regulating intracellular heme B.

A cytochrome *b*_5_-like heme-binding damage resistance protein (Dap) family, including DapA, DapB, and DapC, coordinately regulates the functionality of Erg5 and Erg11 and oppositely affects susceptibility to azoles (39). Biochemical analyses have verified that the free nitrogen of azole molecules is able to compete for the iron atom of the heme in cytochrome P450 enzymes with oxygen to inhibit the synthesis of ergosterol in fungal membranes. Hence, it is conceivable that the *cox7c*-null mutation results in the azole resistance of A. fumigatus through the nonassembly of the cytochrome *c* oxidase complex. Then, the liberation and increase of heme B carrying irons from CYP proteins in the cytoplasm occurs, acting as a substitute for the catalytic heme of cytochrome P450 enzymes to bind the free nitrogen of azole molecules and rescue the synthesis inhibition of ergosterol in fungal membranes.

Reactive oxygen species (ROS) are an important group of free radicals capable of causing direct oxidative damaging effects on DNA, proteins, and lipids, leading to a series of biological consequences (40). In addition to the specific mode of action on ergosterol, antifungal agents generally have ROS-inducing effects in susceptible fungi (41), and they could be scavenged by the appropriate number of certain metabolites (14). Here, Δ*cox7c* showed remarkably reduced ROS production (Fig. 2E and Fig. S3A). This corroborated a previous report in which less ROS was produced in the *cox10* mutants (21). We reasoned that the reduced ROS content alleviates the fungicidal effects of azoles in *cox7c* mutants. Loss of *cox7c* affects normal mitochondrial function, resulting in decreased mitochondrial respiration and less ROS production. Additionally, the superoxide dismutase *sod3* and the putative cytochrome *c* peroxidase *ccp1* genes, which can act as ROS scavengers, were upregulated, as determined by the RNA-seq analysis (Data Set S2) in the *cox7c* mutant. Nevertheless, their effects on ROS clearance are limited.

The *cox7c* deficiency results in different levels of resistance/tolerance to various kinds of antifungals belonging to the triazoles (ITC, VRC, and PSC), polyenes (AmB), allylamines (TBF), nucleoside analogs (5-FC), sphingolipid synthesis inhibitors (MYR), and echinocandins (CAS). The triazoles inhibit ergosterol synthesis through P450 cytochrome-mediated lanosterol demethylation (Cyp51/Erg11), leading to toxic sterol accumulation and cell death. The polyenes are directed against ergosterols to mediate membrane permeabilization, leading to fungal cell death (6). The allylamines inhibit the enzyme squalene epoxidase (Erg1) at the early stage of ergosterol biosynthesis (6). Metabolites of 5-FC can incorporate into RNA, resulting in the disruption of protein synthesis (42). MYR is a specific inhibitor of serine palmitoyltransferase and of ceramide *de novo* synthesis (43). Caspofungin inhibits the enzyme β-(1,3)-glucan synthase of the fungal cell wall (44). Collectively, Cox7c-mediated multiantifungal resistance may involve the following mechanisms (Fig. 6D). First, the cytochrome *c* oxidase has a similar intermediate structure as heme within the P450 enzyme. A *cox7c* deletion in the mitochondria increased the intracellular reductive heme B concentration, to some extent, and it acts as triazole ligands to neutralize fungicides, which is the main reason for the azole resistance of the *cox7c* mutants. Second, the *cox7c* mutation reduced intracellular ROS production, leading to the *cox7c* mutants being more tolerant to AmB and TBF.

## MATERIALS AND METHODS

### Strains, media, and culture conditions.

All the A. fumigatus strains used in this study are shown in Table S1. The media used in this study included rich medium YAG (0.5% yeast extract, 2% glucose, 0.1% 1,000× trace elements, and 2% agar), YUU (YAG supplemented with 5 mM uridine and 10 mM uracil), and minimal medium (MM) (1% glucose, 0.1% 1,000× trace elements, 5% 20× salts [pH 6.5], and 2% agar). For the plate test, conidia of freshly cultured strains were harvested, adjusted to 1 × 10^7^ spores per mL in sterile water, and then inoculated on indicated medium in the absence or presence of different concentrations of ITC, VRC, caspofungin (CAS), posaconazole (PSC), terbinafine (TBF), amphotericin B (AmB), myriocin (MYR), 5-fluorocytosine (5-FC), hemin, sodium nitrite (NaNO_2_), bathophenanthroline disulfonic acid disodium salt hydrate (BPS), or metal ions (FeCl_3_, FeCl_2_, CuSO_4_, MgCl_2_, ZnCl_2_, and CaCl_2_). ITC, VRC, PSC, TBF, AmB, MYR, and hemin were prepared in dimethyl sulfoxide. CAS, 5-FC, BPS, and metal ion compounds were dissolved directly in sterile deionized water.

For cryopreservation time series and azole induction analyses of A. fumigatus A1160^C^ and Af293, storage of a conidial suspension (1 × 10^7^) in sterile deionized water at 4°C or −20°C for 0 to 10 weeks was performed. Then, we spot inoculated 2 μL of conidial suspension on YAG solid medium containing different types of antifungals.

### Next-generation sequencing.

For genome resequencing analysis, spores from A. fumigatus A1160^C^ and mutation strains (IM1 and IM2) were inoculated into YAG liquid medium, respectively, and shaken for 20 h at 37°C at 220 rpm. The harvested mycelia were sent to Shanghai Biotechnology Corporation for DNA extraction and genome resequencing analysis. The library was prepared according to the TruSeq DNA sample preparation guide (Illumina; catalog no. 15026486 Rev. C). The quantity of libraries was assessed by Qubit 2.0 fluorometer. The quality and size of libraries were measured by Agilent 2100 Bioanalyzer high-sensitivity DNA assay according to the reagent kit guide. For Illumina sequencing, the qualified libraries were applied to 2 × 150-bp paired-end sequencing on Illumina HiSeq X Ten platform (Illumina).

FASTQ files were aligned to A. fumigatus A1163 reference genome (GenBank assembly accession no. GCA_000150145) by BWA v0.7.13. The aligned files (SAM/BAM format files) were sorted by SAMtools (v1.3) first, and then duplicates were flagged by using Picard (v2.2.4). By using GATK v3.5, reads were locally realigned, and base qualities were recalibrated. Finally, mapping statistics include coverage and depth and were generated from recalibrated files by BEDTools (v2.16.1) and in-house perl/python scripts. Variants were genotyped from recalibrated BAM files using the multisample processing mode of the UnifiedGenotyper tool from the GATK. Then, variant quality score recalibration (VQSR) was used to reduce false positives of variant calling.

For transcriptome analysis, samples of the A1160^C^ and IM1 strains were collected under the same culture conditions as above. The details of total RNA isolation, library construction, sequencing, and data processing procedures (SRA accession numbers SRX12366462 to SRX12366467) were described in a previous study (14).

### Deletion and complementation of *cox7c*.

All primers used in this study are displayed in Table S2. For deletion of the *cox7c* gene, a fusion PCR approach was employed to generate the fragment sequentially containing the upstream fragment, Neurospora crassa *pyr4*-selectable marker, and downstream fragment (45). Upstream flanking sequences of about 876 bp that corresponded to the region immediately upstream of the *cox7c* start codon were amplified from the A1160 genomic DNA using the primers *cox7c* P1 and *cox7c* P3. Downstream flanking sequences about 932 bp that corresponded to the regions immediately downstream of the *cox7c* stop codon were amplified with the primers *cox7c* P4 and *cox7c* P6. The N. crassa *pyr4* was used as a selectable nutritional marker for transformation and amplified from plasmid pAL5 (45) with the primers pyr4 F and pyr4 R. These three fragments were then mixed as templates and used in a fusion PCR with primers *cox7c* P2 and *cox7c* P5 to construct the *cox7c* deletion cassette. Then, the *cox7c* deletion cassette was transformed into the wild-type A1160 strain to achieve homologous recombination. A diagnostic PCR assay was performed to identify the absence of the *cox7c* gene with the primers *cox7c* P2 and Diag *cox7c*. For the complementation of the *cox7c* deletion mutant, the full-length *cox7c* gene was amplified using the primer pair *cox7c*-com S1/S2, which contains the native promoter, 5′-untranslated region (5′-UTR), gene coding, and 3′-UTR sequences. The complementation cassette was subsequently transformed into the PAN7-1 plasmid. The recombinant plasmid was then transformed into the *cox7c* deletion strain, and transformants were selected on YAG medium supplemented with 200 μg/mL hygromycin (46).

### Generation of the *Tet-hem15* mutant.

For *Tet-hem15* mutant construction, the promoter of *hem15* was replaced with a conditional doxycycline-inducible *tet-on* promoter (46), and the selection marker pyrithiamine resistance cassette was replaced by *pyr4*. First, the selective marker *pyr4* and the *tet-on* promoter were amplified from the pAL5 and pCH008 plasmids using the primer pairs pyr4 F/R and tet F/R, respectively. Approximately 1-kb areas of the upstream and downstream flanking sequences of the *hem15* 5′-UTR region were amplified with the primer pairs tet-hem15 P1/P3 and tet-hem15 P4/P6, respectively. The resulting fragments were then cloned into pCE-Zero vector using the pEASY-Basic seamless cloning and assembly kit (TransGen Biotech), yielding pCE-Zero-hem15. The fragment amplified from pCE-Zero-hem15 using tet-hem15 P2/P5 was transformed into the A1160 strain to generate the *Tet-hem15* mutant. Transformants were selected on medium without uracil and uridine.

### Antifungal susceptibility testing.

The conidia of tested Aspergillus strains were harvested, adjusted to 1 × 10^7^ spores per mL in phosphate-buffered saline, and then inoculated into RPMI 1640 liquid medium in the presence of different concentrations of antifungal drugs (ITC, VRC, PSC, TBF, AmB, MYR, or 5-FC). Broth microdilution antimicrobial susceptibility testing was performed according to CLSI M38-A2 (27). Briefly, 2-fold serial drug dilutions were prepared in flat-bottom 96-well microtiter plates (100 μL per well), and drug-free wells were used as control. Each well was inoculated with 100 μL of freshly isolated spores (1 × 10^4^ conidia per mL) suspended in RPMI 1640 media. After 48 h of incubation at 35°C, the MIC data were recorded as the lowest drug concentration at which no growth was observed. These MIC assays were repeated four times.

### Intracellular drug detection.

The intracellular drug was measured as described previously (47). Briefly, 1 × 10^7^ spores of the indicated strains were cultured in 100 mL of liquid YAG medium at 37°C and shaken at 220 rpm for 20 h. Then, a final concentration of 1 μg/mL of ITC was added to the media and continued to culture for 2 h. Mycelia were harvested and washed with distilled water to remove the extracellular ITC and were then lyophilized. Approximately 100 mg of lyophilized mycelia was incubated in 2 mL of 50% (vol/vol) methanol in water and homogenized using ceramic beads. The cell debris and ceramic beads were then removed by centrifugation at 13,000 × *g* for 10 min. After which, the supernatant was analyzed using HPLC on an AQ C_18_ column (250 mm by 4.6 mm; 5 μm) with an isocratic profile at 65% (vol/vol) acetonitrile in phosphate buffer at a flow rate of 1 mL/min. The peak corresponding to ITC was detected using absorption spectrophotometry at a wavelength of 265 nm. The results indicate that the retention time for ITC was 8.10 min. The standard curve was prepared with 0.1, 0.5, 1, 5, and 10 μg/mL of ITC. The detectable concentration range of ITC in samples is 1 to 4 μg/mL. The final concentration unit of ITC in the samples was converted to micrograms per gram (drug weight per hypha weight).

### Intracellular ROS detection.

Intracellular reactive oxygen species (ROS) were measured as described previously (14). In brief, spores (1 × 10^5^) of the indicated strains were cultured in 200 μL of liquid YAG medium in the black and clear bottom of the 96-well Costar plate at 37°C for 10 h. After the culture was completed, hyphae were washed in PBS three times. 2,7-Dichlorofuorescin diacetate (DCFH-DA) was added at a final concentration of 15 μM and then incubated at 37°C for 30 min without light. Then, hyphae were washed in PBS three times, and 200 μL of 1 μg/mL of ITC or 1.5 mM H_2_O_2_ in PBS was added to the dish, followed by incubation at 37°C for 3 h. The fluorescence intensity was measured with an excitation filter at 495 nm and an emission filter at 530 nm in a microtiter plate reader (Infinite M200 Pro; Tecan, Switzerland) at 37°C. Unstained cells were used as a blank.

### Ergosterol detection.

Ergosterol in A. fumigatus strains was extracted as described previously with slight modifications (48). Spores (1 × 10^7^) of the indicated strains were cultured in 100 mL of liquid YAG medium at 37°C and shaken at 220 rpm for 20 h. The mycelia were harvested by filtration, and 200 mg of dry mycelia was treated with 3 mL of a 25% alcohol potassium hydroxide solution (3:2 dilution of methanol/ethanol), followed by vortexing for 1 min. After incubation at 85°C for 1 h, the samples were treated with 1 mL of distilled water and 3 mL of hexane and vortexed for 3 min. The upper hexane layer was transferred, blown dry by nitrogen, and solubilized in 1 mL methanol. Before HPLC analysis, the samples were filtered through 0.45-μm-pore-size filters. Under an isocratic profile at 97% (vol/vol) methanol at a 1-mL/min flow rate, the total ergosterol was detected at 280 nm on an AQ C_18_ column (250 mm by 4.6 mm; 5 μm). The retention time for ergosterol was 9.50 min, and the standard curve was prepared with 0.025, 0.05, 0.1, 0.4, and 0.8 mg/mL of ergosterol. The detectable concentration range of ergosterol in the samples was 0.08 to 0.16 mg/mL. The concentration unit of ergosterol in the samples was converted to milligrams per gram (drug weight per hypha weight).

### Cellular iron content detection.

Cellular iron content was determined according to the BPS-based colorimetric method (49). Spores (1 × 10^7^) of the indicated strains were cultured in 100 mL of liquid YAG medium at 37°C and shaken at 220 rpm for 20 h. The mycelia were harvested by filtration, and 10 mg of dry mycelia was treated with 500 μL of 3% nitric acid and incubated at 95°C for 2 h to digest the cells completely. We then mixed 200 μL of cell supernatant with 80 μL of 38 mg/mL sodium ascorbate, 160 μL of 1.7 mg/mL BPS, and 63 μL of 4 M ammonium acetate. After 10 min incubation at room temperature, the optical density at 535 nm (OD_535_) of the BPS-Fe complex was determined by a microtiter plate reader (Infinite M200 Pro; Tecan, Switzerland). The OD_680_ was also recorded as the nonspecific absorbance. Experiments were performed in triplicate, and the iron content was calculated as the following formula: (OD_535_ − OD_680_)/hypha weight, and displayed in arbitrary units (AU).

### Heme detection.

Intracellular heme was prepared and analyzed by HPLC as described elsewhere with a minor modification (31, 50). The total hemin was extracted from 20 mg of A. fumigatus dry mycelia with 0.4 mL of acetone containing 2.5% HCl. The mixture was vortexed, disrupted by ultrasonication for 10 min, centrifuged for 5 min at 15,000 × *g*, and mixed with 0.6 mL of 50% acetonitrile. Insoluble material was removed, and the extract was clarified by second centrifugation and applied to an AQ C_18_ column (250 mm by 4.6 mm; 5 μm). Hemin was eluted at a flow rate of 1 mL/min using a 30% to 50% acetonitrile gradient over the first 5 min, followed by a 50% to 75% linear acetonitrile gradient over the subsequent 35 min, and, finally, 50% acetonitrile gradient over the last 5 min. All gradient solutions contained 0.05% trifluoroacetic acid. The elution of heme compounds was monitored at 400 nm. The retention time of the products was 17.5 min (heme B), 35 min, and 36.5 min. The standard curve was prepared with 0.5, 1, 5, 10, and 20 μg/mL of hemin. The detectable concentration range of heme B in samples was 0.5 to 16 μg/mL. The final concentration unit of heme B in the samples was converted to microgram per gram (drug weight per hypha weight).

### Data availability.

The raw Illumina sequencing data were deposited in SRA at NCBI with accession numbers SRX12366077 to SRX12366079.

## References

[1] Latge JP, Chamilos G. 2019. *Aspergillus fumigatus* and Aspergillosis in 2019. Clin Microbiol Rev 33:e00140-18. 10.1128/CMR.00140-18. 31722890 PMC6860006

[2] Cheng MP, Orejas JL, Arbona-Haddad E, Bold TD, Solomon IH, Chen KW, Pandit A, Kusztos AE, Cummins KC, Liakos A, Marty FM, Koo S, Hammond SP. 2020. Use of triazoles for the treatment of invasive aspergillosis: a three-year cohort analysis. Mycoses 63:58–64. 10.1111/myc.13013. 31587405

[3] Mousset S, Buchheidt D, Heinz W, Ruhnke M, Cornely OA, Egerer G, Kruger W, Link H, Neumann S, Ostermann H, Panse J, Penack O, Rieger C, Schmidt-Hieber M, Silling G, Sudhoff T, Ullmann AJ, Wolf HH, Maschmeyer G, Bohme A. 2014. Treatment of invasive fungal infections in cancer patients-updated recommendations of the Infectious Diseases Working Party (AGIHO) of the German Society of Hematology and Oncology (DGHO). Ann Hematol 93:13–32. 10.1007/s00277-013-1867-1. 24026426 PMC3889633

[4] Garcia-Rubio R, Cuenca-Estrella M, Mellado E. 2017. Triazole resistance in Aspergillus species: an emerging problem. Drugs 77:599–613. 10.1007/s40265-017-0714-4. 28236169

[5] Kelly SL, Lamb DC, Baldwin BC, Corran AJ, Kelly DE. 1997. Characterization of Saccharomyces cerevisiae CYP61, sterol delta22-desaturase, and inhibition by azole antifungal agents. J Biol Chem 272:9986–9988. 10.1074/jbc.272.15.9986. 9092539

[6] White TC, Marr KA, Bowden RA. 1998. Clinical, cellular, and molecular factors that contribute to antifungal drug resistance. Clin Microbiol Rev 11:382–402. 10.1128/CMR.11.2.382. 9564569 PMC106838

[7] Perlin DS, Rautemaa-Richardson R, Alastruey-Izquierdo A. 2017. The global problem of antifungal resistance: prevalence, mechanisms, and management. Lancet Infect Dis 17:e383–e392. 10.1016/S1473-3099(17)30316-X. 28774698

[8] Van Daele R, Spriet I, Wauters J, Maertens J, Mercier T, Van Hecke S, Bruggemann R. 2019. Antifungal drugs: what brings the future? Med Mycol 57:S328–S343. 10.1093/mmy/myz012. 31292663

[9] Sugui JA, Kwon-Chung KJ, Juvvadi PR, Latge JP, Steinbach WJ. 2014. Aspergillus fumigatus and related species. Cold Spring Harb Perspect Med 5:a019786. 10.1101/cshperspect.a019786. 25377144 PMC4315914

[10] Hokken MWJ, Zoll J, Coolen JPM, Zwaan BJ, Verweij PE, Melchers WJG. 2019. Phenotypic plasticity and the evolution of azole resistance in Aspergillus fumigatus; an expression profile of clinical isolates upon exposure to itraconazole. BMC Genomics 20:28. 10.1186/s12864-018-5255-z. 30626317 PMC6327609

[11] Rybak JM, Fortwendel JR, Rogers PD. 2019. Emerging threat of triazole-resistant Aspergillus fumigatus. J Antimicrob Chemother 74:835–842. 10.1093/jac/dky517. 30561652 PMC6657284

[12] Jeanvoine A, Rocchi S, Bellanger AP, Reboux G, Millon L. 2020. Azole-resistant Aspergillus fumigatus: a global phenomenon originating in the environment? Med Mal Infect 50:389–395. 10.1016/j.medmal.2019.07.014. 31472992

[13] Wei X, Zhang Y, Lu L. 2015. The molecular mechanism of azole resistance in Aspergillus fumigatus: from bedside to bench and back. J Microbiol 53:91–99. 10.1007/s12275-015-5014-7. 25626363

[14] Chen M, Zhong G, Wang S, Zhu J, Tang L, Li L. 2020. tpo3 and dur3, Aspergillus fumigatus plasma membrane regulators of polyamines, regulate polyamine homeostasis and susceptibility to itraconazole. Front Microbiol 11:563139. 10.3389/fmicb.2020.563139. 33391196 PMC7772357

[15] Kowalski CH, Morelli KA, Schultz D, Nadell CD, Cramer RA. 2020. Fungal biofilm architecture produces hypoxic microenvironments that drive antifungal resistance. Proc Natl Acad Sci USA 117:22473–22483. 10.1073/pnas.2003700117. 32848055 PMC7486789

[16] Garcia-Rubio R, Alcazar-Fuoli L, Monteiro MC, Monzon S, Cuesta I, Pelaez T, Mellado E. 2018. Insight into the significance of Aspergillus fumigatus cyp51A polymorphisms. Antimicrob Agents Chemother 62:e00241-18. 10.1128/AAC.00241-18. 29632011 PMC5971592

[17] Handelman M, Meir Z, Scott J, Shadkchan Y, Liu W, Ben-Ami R, Amich J, Osherov N. 2021. Point mutation or overexpression of Aspergillus fumigatus cyp51B, encoding lanosterol 14alpha-sterol demethylase, leads to triazole resistance. Antimicrob Agents Chemother 65:e0125221. 10.1128/AAC.01252-21. 34310208 PMC8448118

[18] Toyotome T, Onishi K, Sato M, Kusuya Y, Hagiwara D, Watanabe A, Takahashi H. 2021. Identification of novel mutations contributing to azole tolerance of Aspergillus fumigatus through in vitro exposure to tebuconazole. Antimicrob Agents Chemother 65:e0265720. 10.1128/AAC.02657-20. 34125587 PMC8370235

[19] Gsaller F, Hortschansky P, Furukawa T, Carr PD, Rash B, Capilla J, Muller C, Bracher F, Bowyer P, Haas H, Brakhage AA, Bromley MJ. 2016. Sterol biosynthesis and azole tolerance is governed by the opposing actions of SrbA and the CCAAT binding complex. PLoS Pathog 12:e1005775. 10.1371/journal.ppat.1005775. 27438727 PMC4954732

[20] Hagiwara D, Arai T, Takahashi H, Kusuya Y, Watanabe A, Kamei K. 2018. Non-cyp51A azole-resistant Aspergillus fumigatus isolates with mutation in HMG-CoA reductase. Emerg Infect Dis 24:1889–1897. 10.3201/eid2410.180730. 30226177 PMC6154143

[21] Li Y, Zhang Y, Zhang C, Wang H, Wei X, Chen P, Lu L. 2020. Mitochondrial dysfunctions trigger the calcium signaling-dependent fungal multidrug resistance. Proc Natl Acad Sci USA 117:1711–1721. 10.1073/pnas.1911560116. 31811023 PMC6983421

[22] Song J, Zhou J, Zhang L, Li R. 2020. Mitochondria-mediated azole drug resistance and fungal pathogenicity: opportunities for therapeutic development. Microorganisms 8:1574. 10.3390/microorganisms8101574. PMC7600254 33066090

[23] Shekhova E, Kniemeyer O, Brakhage AA. 2017. Induction of mitochondrial reactive oxygen species production by itraconazole, terbinafine, and amphotericin B as a mode of action against Aspergillus fumigatus. Antimicrob Agents Chemother 61:e00978-17. 10.1128/AAC.00978-17. 28848005 PMC5655112

[24] Neubauer M, Zhu Z, Penka M, Helmschrott C, Wagener N, Wagener J. 2015. Mitochondrial dynamics in the pathogenic mold Aspergillus fumigatus: therapeutic and evolutionary implications. Mol Microbiol 98:930–945. 10.1111/mmi.13167. 26272083

[25] Herrmann JM, Funes S. 2005. Biogenesis of cytochrome oxidase-sophisticated assembly lines in the mitochondrial inner membrane. Gene 354:43–52. 10.1016/j.gene.2005.03.017. 15905047

[26] Sharma C, Nelson-Sathi S, Singh A, Pillai MR, Chowdhary A. 2019. Genomic perspective of triazole resistance in clinical and environmental Aspergillus fumigatus isolates without cyp51A mutations. Fungal Genet Biol 132:103265. 10.1016/j.fgb.2019.103265. 31465846

[27] Clinical and Laboratory Standards Institute. 2008. Reference method for broth dilution antifungal susceptibility testing of filamentous fungi, 2nd ed. CLSI M38-A2. Clinical and Laboratory Standards Institute, Wayne, PA.

[28] Timmons AJ, Symes MD. 2015. Converting between the oxides of nitrogen using metal-ligand coordination complexes. Chem Soc Rev 44:6708–6722. 10.1039/c5cs00269a. 26158348

[29] Gebicka L. 2020. Redox reactions of heme proteins with flavonoids. J Inorg Biochem 208:111095. 10.1016/j.jinorgbio.2020.111095. 32442763

[30] Nobrega MP, Graminha MA, Troitskaya EN, Nobrega FG. 1998. Study of a region on yeast chromosome XIII that complements pet G199 mutants (COX7) and carries a new non-essential gene. Braz J Med Biol Res 31:355–363. 10.1590/s0100-879x1998000300004. 9698782

[31] Zhou S, Narukami T, Nameki M, Ozawa T, Kamimura Y, Hoshino T, Takaya N. 2012. Heme-biosynthetic porphobilinogen deaminase protects Aspergillus nidulans from nitrosative stress. Appl Environ Microbiol 78:103–109. 10.1128/AEM.06195-11. 22038601 PMC3255638

[32] Juckett M, Zheng Y, Yuan H, Pastor T, Antholine W, Weber M, Vercellotti G. 1998. Heme and the endothelium. Effects of nitric oxide on catalytic iron and heme degradation by heme oxygenase. J Biol Chem 273:23388–23397. 10.1074/jbc.273.36.23388. 9722574

[33] Waheed SM, Ghosh A, Chakravarti R, Biswas A, Haque MM, Panda K, Stuehr DJ. 2010. Nitric oxide blocks cellular heme insertion into a broad range of heme proteins. Free Radic Biol Med 48:1548–1558. 10.1016/j.freeradbiomed.2010.02.038. 20211245 PMC2866197

[34] Hoffman M, Gora M, Rytka J. 2003. Identification of rate-limiting steps in yeast heme biosynthesis. Biochem Biophys Res Commun 310:1247–1253. 10.1016/j.bbrc.2003.09.151. 14559249

[35] Qian W, Ma D, Xiao C, Wang Z, Zhang J. 2012. The genomic landscape and evolutionary resolution of antagonistic pleiotropy in yeast. Cell Rep 2:1399–1410. 10.1016/j.celrep.2012.09.017. 23103169 PMC3513580

[36] Long N, Xu X, Qian H, Zhang S, Lu L. 2016. A putative mitochondrial iron transporter MrsA in Aspergillus fumigatus plays important roles in azole-, oxidative stress responses and virulence. Front Microbiol 7:716. 10.3389/fmicb.2016.00716. 27433157 PMC4922219

[37] Blatzer M, Barker BM, Willger SD, Beckmann N, Blosser SJ, Cornish EJ, Mazurie A, Grahl N, Haas H, Cramer RA. 2011. SREBP coordinates iron and ergosterol homeostasis to mediate triazole drug and hypoxia responses in the human fungal pathogen Aspergillus fumigatus. PLoS Genet 7:e1002374. 10.1371/journal.pgen.1002374. 22144905 PMC3228822

[38] Travassos LH, Vasconcellos LR, Bozza MT, Carneiro LA. 2017. Heme and iron induce protein aggregation. Autophagy 13:625–626. 10.1080/15548627.2016.1271515. 28055290 PMC5361590

[39] Song J, Zhai P, Zhang Y, Zhang C, Sang H, Han G, Keller NP, Lu L. 2016. The Aspergillus fumigatus damage resistance protein family coordinately regulates ergosterol biosynthesis and azole susceptibility. mBio 7:e01919-15. 10.1128/mBio.01919-15. 26908577 PMC4791848

[40] Tang Y, Guo Y, Zhang L, Cai J, Yang P. 2014. A novel electrochemical biosensor for monitoring protein nitration damage affected by NaNO2/hemin/H2O2. Biosens Bioelectron 54:628–633. 10.1016/j.bios.2013.11.052. 24333935

[41] Delattin N, Cammue BP, Thevissen K. 2014. Reactive oxygen species-inducing antifungal agents and their activity against fungal biofilms. Future Med Chem 6:77–90. 10.4155/fmc.13.189. 24358949

[42] Gsaller F, Furukawa T, Carr PD, Rash B, Jochl C, Bertuzzi M, Bignell EM, Bromley MJ. 2018. Mechanistic basis of pH-dependent 5-flucytosine resistance in Aspergillus fumigatus. Antimicrob Agents Chemother 62:e02593-17. 10.1128/AAC.02593-17. 29610197 PMC5971587

[43] Wadsworth JM, Clarke DJ, McMahon SA, Lowther JP, Beattie AE, Langridge-Smith PR, Broughton HB, Dunn TM, Naismith JH, Campopiano DJ. 2013. The chemical basis of serine palmitoyltransferase inhibition by myriocin. J Am Chem Soc 135:14276–14285. 10.1021/ja4059876. 23957439

[44] Nevez G, Le Gal S. 2019. Caspofungin and pneumocystis pneumonia: it is time to go ahead. Antimicrob Agents Chemother 63:e01296-19. 10.1128/AAC.01296-19. 31548210 PMC6761523

[45] Jiang H, Shen Y, Liu W, Lu L. 2014. Deletion of the putative stretch-activated ion channel Mid1 is hypervirulent in Aspergillus fumigatus. Fungal Genet Biol 62:62–70. 10.1016/j.fgb.2013.11.003. 24239700

[46] Guan L, Lu R, Wu Z, Zhong G, Zhang S. 2020. Precise expression of Afmed15 is crucial for asexual development, virulence, and Survival of Aspergillus fumigatus. mSphere 5:e00771-20. 10.1128/mSphere.00771-20. PMC7568654 33028685

[47] Long N, Zeng L, Qiao S, Li L, Zhong G. 2018. Aspergillus fumigatus Afssn3-Afssn8 Pair reverse regulates azole resistance by conferring extracellular polysaccharide, sphingolipid pathway intermediates, and efflux pumps to biofilm. Antimicrob Agents Chemother 62:e01978-17. 10.1128/AAC.01978-17. 29311083 PMC5826118

[48] Wei X, Chen P, Gao R, Li Y, Zhang A, Liu F, Lu L. 2017. Screening and characterization of a non-cyp51A mutation in an Aspergillus fumigatus cox10 strain conferring azole resistance. Antimicrob Agents Chemother 61:e02101-16. 10.1128/AAC.02101-16. 27799210 PMC5192120

[49] Jia C, Zhang K, Yu QL, Zhang B, Xiao CP, Dong YJ, Chen YL, Zhang BA, Xing LJ, Li MC. 2015. Tfp1 is required for ion homeostasis, fluconazole resistance and N-acetylglucosamine utilization in Candida albicans. Biochim Biophys Acta 1853:2731–2744. 10.1016/j.bbamcr.2015.08.005. 26255859

[50] Antonicka H, Mattman A, Carlson CG, Glerum DM, Hoffbuhr KC, Leary SC, Kennaway NG, Shoubridge EA. 2003. Mutations in COX15 produce a defect in the mitochondrial heme biosynthetic pathway, causing early-onset fatal hypertrophic cardiomyopathy. Am J Hum Genet 72:101–114. 10.1086/345489. 12474143 PMC378614

