# Supplementary material for: Deletion of cox7c Results in Pan-Azole Resistance in Aspergillus fumigatus
Source: Antimicrob Agents Chemother. 2022 Jun 1;66(6):e00151-22. doi: 10.1128/aac.00151-22 (PMC9211413; doi:10.1128/aac.00151-22)
Supplement: Supplemental file 3 — Fig. S1 to S4 and Tables S1 and S2. Download aac.00151-22-s0003.pdf, PDF file, 1.5 MB [file aac.00151-22-s0003.pdf]

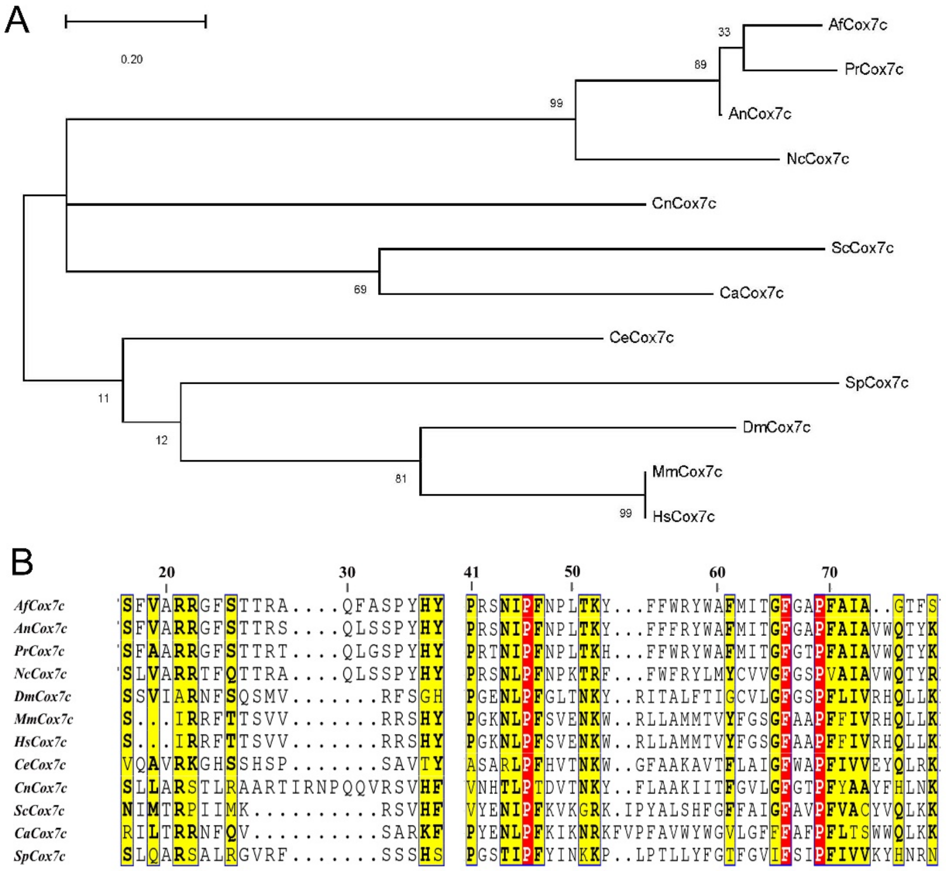

FIG S1 Phylogenetic and identity analysis of Cox7c-like family for selected organisms. (A) Phylogenetic tree representing the evolutionary relationships between Cox7c orthologs. The evolutionary tree was constructed using MEGA-X maximum-likelihood analyses from amino acid sequences of following Cox7c orthologs with their accession numbers: AfCox7c (*Aspergillus fumigatus*, EDP50092.1), PrCox7c (*Penicillium rubens*, XP_002565739.1), AnCox7c (*Aspergillus nidulans*, CBF77578.1), NcCox7c (*Neurospora crassa*, XP_956449.1), CnCox7c (*Cryptococcus neoformans*, OXM75973.1), ScCox7c/8 (*Saccharomyces cerevisiae*, AJV66559.1), CaCox7c (*Candida albicans*, KGR13581.1), CeCox7c (*Caenorhabditis elegans*, NP_492596.2)

SpCox7c/8 (*Schizosaccharomyces pombe*, Q9P4W1.1), DmCox7c (*Drosophila melanogaster*, XP_001360701.1), MmCox7c (*Mus musculus*, NP_031775.1), and HsCox7c (*Homo sapiens*, AAP97260.1). (B) Comparison of amino acid sequence identities between Cox7c in *A. fumigatus* and Cox7c-like family in selected organisms. Sequences were obtained from the NCBI protein database. Multiple-sequence alignment was performed by using MAFFT (https://mafft.cbrc.jp/alignment/server/index.html).

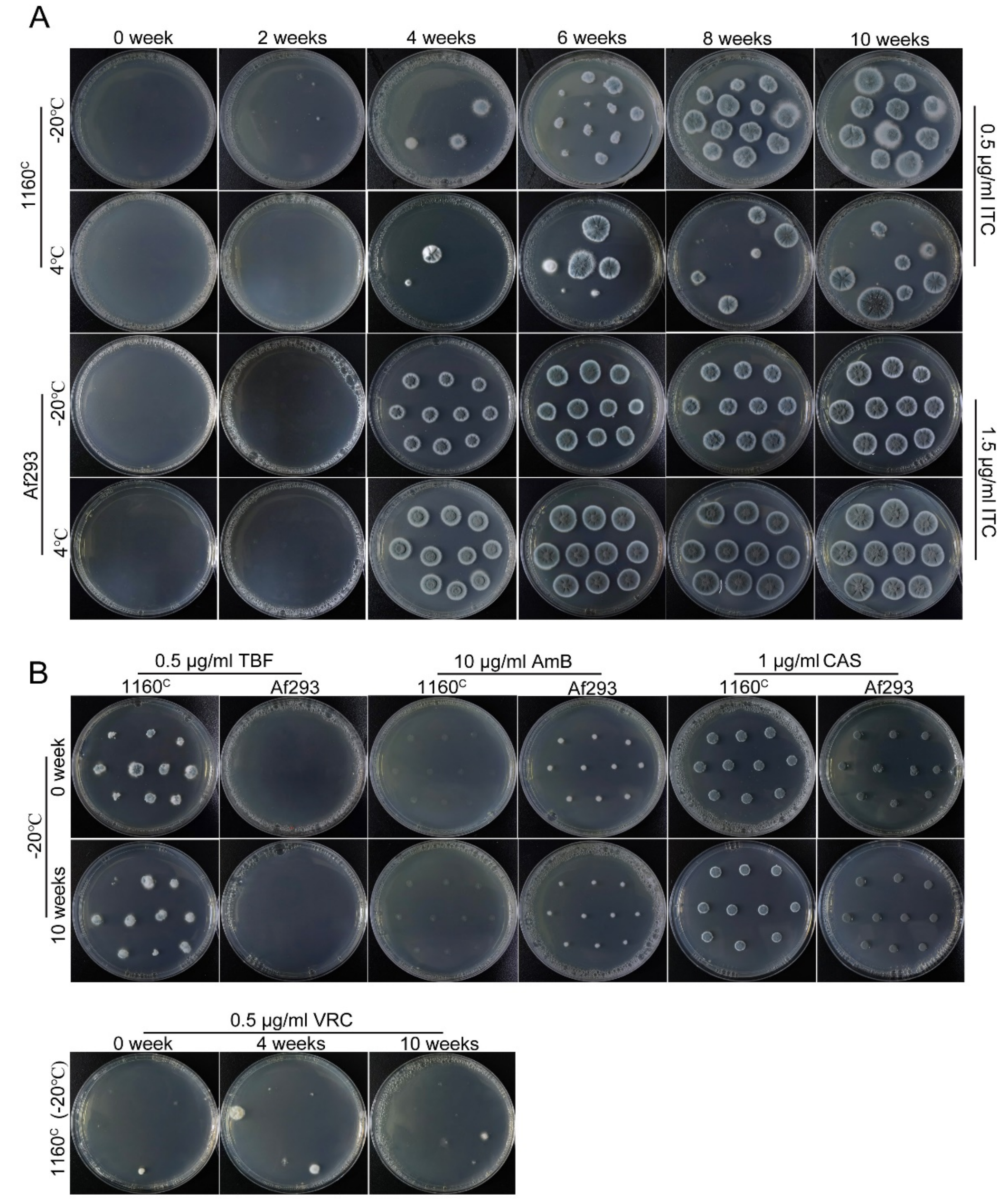

FIG S2 Long term cryopreservation of *A. fumigatus* wild-type spores leads to ITC tolerance. (A) *A. fumigatus* A1160^{C} or Af293 spores suffering different cryopreservation times were cultured on YAG agar plates supplemented with 0.5 or 1.5 µg/ml ITC at 37℃ for 4 days. Tested spores were cryopreserved in sterile deionized water at -20℃ or 4℃ for 0 to 10 weeks. (B) *A. fumigatus* A1160^{C} or Af293 spores

cryopreserved in sterile deionized water at -20°C for 0 or 10 weeks were cultured on YAG agar plates supplemented with 0.5 µg/ml VRC, 0.5 µg/ml TBF, 10 µg/ml AmB or 1 µg/ml CAS at 37°C. The plates supplemented with TBF, AmB or CAS were incubated for 4, 2, and 2 days, respectively.

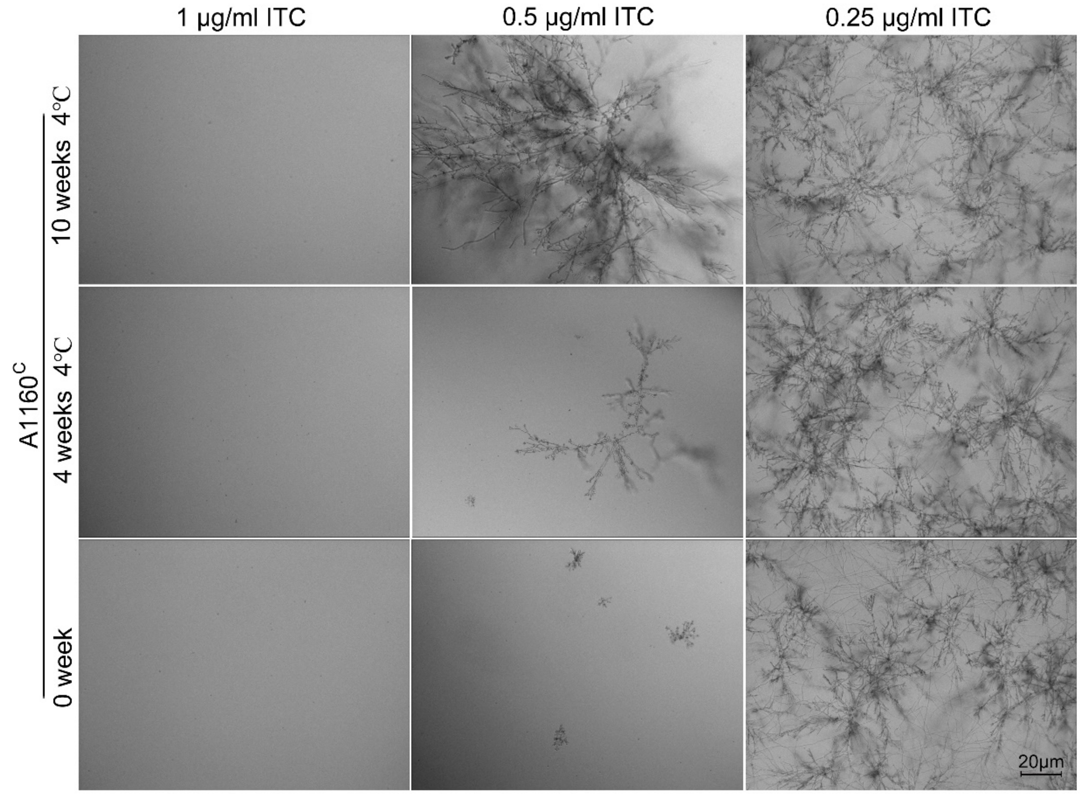

FIG S3 Microdilution assay for A1160^{C} conidia treated under different conditions. The MICs of fresh prepared conidia (0 week), 4, and 10 weeks cold-stored (4°C) conidia were all 1 µg/ml. Note the cold-stored conidia displayed accelerated hyphal growth (ITC tolerance) compared to fresh prepared conidia in the presence of 0.5 µg/ml ITC. Growth is shown after 48 h at 35°C in RPMI 1640 media. MIC values were determined by microscopy. Bars, 20 µm.

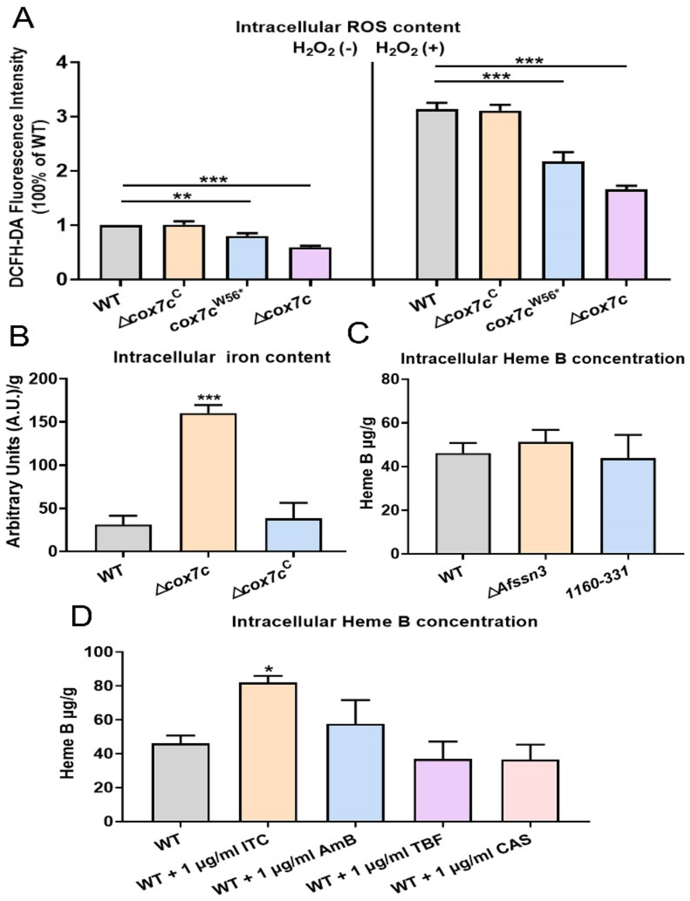

FIG S4 Analyses of intracellular ROS, iron, and heme B in the displayed strains under indicated treatment conditions. (A) Measurement for ROS production in the WT (A1160^{C}), Δ*cox7c*, and Δ*cox7c*^{C} strains with or without 1.5 mM H_2O_2 stimulus. (B) The total iron levels in the indicated strains were measured by the BPS-based colorimetric method. (C) HPLC analyses of the intracellular concentrations of heme B in the displayed strains. (D) Intracellular concentrations of heme in the WT (A1160^{C}) strain stimulated by different type of antifungals. The values of ROS fluorescence intensity, iron content, and heme B concentration were analyzed by one-way ANOVA with unpaired Student *t*-test and presented as the means ± SD of three biological samples. Error bars represent the corresponding standard deviations. *$P < 0.05$; **$P < 0.01$; ***$P < 0.001$.

TABLE S1 *Aspergillus fumigatus* strains used in this study.

**Strain**	**Genotype**	**Reference**
A1160	Δ*KU80*, *pyrG1*	FGSC
A1160^{C}	Δ*KU80*, *A1160::pyrG1*	This study
Δ*cox7c*	Δ*KU80*, *pyrG1*, Δ*cox7c::pyr4*	This study
Δ*cox7c*^{C}	Δ*KU80*, *pyrG1*, Δ*cox7c::cox7c*	This study
IM1	Δ*KU80*, *A1160::pyrG1*, *cox7c*^{W56*}	This study
IM2	Δ*KU80*, *A1160::pyrG1*, *cox7c*^{W56*}	This study
Tet-hem15	*Tet(p)::hem15::pyr4;Δku80;A1160::pyrG1*	This study
Δ*Afssn3*	*ΔKU80*, *pyrG1*, *ΔAfssn3::pyr4*	(1)
1160-331(*cyp51A*^{G54W})	*ΔKU80*, *pyrG1*, *cyp51A*^{G54W}*::pyrG1*	(2)

TABLE S2 All primers used in this study.

Primer name	Primer sequence 5’-3’ (used for deletion and complementation)
cox7c P1	ACTCCGCAACTACCGTCTTAC
cox7c P2	GTTCTCAGGCGGCTTGTG
cox7c P3	GGCTTGTCTGCTCCCGCGATACCGTCGAAGTAACCCG
cox7c P4	ACGCCAGGGTTTTCCCTAGCGGATTGTGGTAGATGGG
cox7c P5	ATCTTCGGGGGTGAAGGTG
cox7c P6	CGTCATCGTTGCTGCTCAATC
Diagcox7c	TACCAGCAATGGCGAAGGG
cox7c-com S1	ACTCCGCAACTACCGTCTTAC
cox7c-com S2	CGTCATCGTTGCTGCTCAATC
tet-hem15-P1	TTGCTTTGCCTTCGTCCTCC
tet-hem15-P2	AAAGGTAGCAGTGAGGATACGATAG
tet-hem15-P3	CGATTAAGTTGGGTAACGCCAGGGGAGGTGCGATGATAGTGTA
tet-hem15-P4	CCGCTTGAGCAGACATCACCATGGCTCTCCGCCGGCCATT
tet-hem15-P5	CTGAGAACAAGAGAACGACACC
tet-hem15-P6	TAGGAAATGTGTTAGCGAGCAAATC
tet-F	TGGCGTTACCCAACTTAATCG
tet-R	GGTGATGTCTGCTCAAGCGG
pyr4 F	TGGCGTTACCCAACTTAATCG
pyr4 R	GCTTTCGGGAACTGGCTACTTAT

Primer name	Primer sequence 5'-3' (used for single gene sequencing)
cox7c-F	CGGGTGGTCCATTATCGGG
cox7c-R	TCTAACGGGCATCAACCCATC
cyp51A-SQ1F	TTTCCCACCACTGTAGTTGCTT
cyp51A-SQ2F	GGTGGACGCAGAGACTTGTA
cyp51A-SQ3F	TCGAAATGGTGCCG
cyp51A-SQ4F	ACAAGGGCTTTACTCC
cyp51A-R	TCCCTACCGAACCAGTGGCAAA
erg3-SQ1F	AGGGGTTGGCATCGTCG
erg3-SQ2F	CGTTGTCAACTGCTGCAGTA
erg3-SQ3F	TCACAGGGCTTTCGGTCTTC
erg3-SQ4F	CTACGGGCAGTTTACCACGT
erg3-R	GCGCCTTCCACCAACATC
erg5-SQ1F	CTCCGGCTGTACTACCCG
erg5-SQ2F	TTGGTCTTCCGGTCACACAG
erg5-SQ3F	ACGAGACCGGCACATGAATT
erg5-SQ4F	CGGCCATCAAGAAGATTGCG
erg5-R	GTCGACCAAGACATCAAGGC
erg6-SQ1F	CGCAGACGGAAGGGAAGTC
erg6-SQ2F	CCGCCTCCTTTAACGATCGA
erg6-SQ3F	TCATTCCACTTCTGCCGCTT

erg6-R	ACGGTTAGACAGCCACGTC
srbA-SQ1F	GCGCGATATCCTGACGTTG
srbA-SQ2F	CTTTTTGCCTTGCACGCTCT
srbA-SQ3F	CGGGCGGAGATCTGAATACC
srbA-SQ4F	GAGAGCCCCTACTCGCAATC
srbA-SQ5F	AAAGCCGTCTGGTCCTTACG
srbA-R	CCTCGATCCAGACCAGATGC
hapX-SQ1F	GAGTCGATCAAGCTGAAGCG
hapX-SQ2F	TCTCTCCTCGGCTTTTGCTC
hapX-SQ3F	AAATCCACATTGCGGCGTTC
hapX-SQ4F	CCAATTCACACCACCCCCTT
hapX-R	TCACGAGTCCGTTTGGGTATC
hapX-SQ1F	GACAGCACAGACTTCGCATG
hapX-SQ2F	CTCCAAAGTCAGTCCATTCGC
hapX-SQ3F	TCACCCTGCCCACCAAGGCTC
hapX-SQ4F	TCAACCCGTGCTCAATCATG
hapX-R	CACACATCGCTTGCTACGAG
hapC-SQ1F	CAACCGCCCATCTCTCATTC
hapC-SQ2F	ACCTGTCGCTTCTTTCCTCG
hapC-SQ3F	GAAACCCTCATTTGCCTGCG
hapC-R	CAAGGGTAGCACCGTCCAG

hapE-SQ1F	GCGAGTGGCCATTGTAAGC
hapE-SQ2F	TATTCACAGGTCCCGCAACC
hapE-SQ3F	TGCGAAAGGCTGCGATATCT
hapE-R	CTGCAAACCACTCTGCCTG
hmg1A-SQ1F	GTGGCTCTGTCAATGAATGCAC
hmg1A-SQ2F	GCGCCAGTGTCCGGAATATA
hmg1A-SQ3F	CGAGTTTGCTGGAAGGGAGT
hmg1A-SQ4F	TGGCTGATAGACGTCTTGGC
hmg1A-SQ5F	CTGGAAATCACGCGCATCAG
hmg1A-SQ6F	GACCATGTCAGGACCTAGCG
hmg1A-SQ7F	GGCACGTACCTCTATGTCCG
hmg1A-SQ8F	CTGGCCTGGGCTGATCATTT
hmg1A -R	GATTGGGCGTCCCAGAGG
hmg1B-SQ1F	GTCGTCTTGTGCCTGTTCAC
hmg1B-SQ2F	ATTTCCATCTTCCCCAGCGG
hmg1B-SQ3F	TTCGACAGCGTCAGGAACTC
hmg1B-SQ4F	TCCAGCAGTGTTCGCAGATT
hmg1B-SQ5F	TCGCTGGAGCATTAAGGAGC
hmg1B-SQ6F	TTTCCCTGCGAGGCAAGATT
hmg1B-SQ7F	CCACGTTATGGCGACTGAGT
hmg1B -R	TCAGAGCTTACCACCTAGCTG

cox10-SQ1F	GATTTGAGGAGCCTTGC
cox10-SQ2F	GATCGAGGAGGAGGGCGC
cox10-SQ3F	CCGATCATGAATCAACTCCC
cox10-SQ4F	GAACCTAAATACGATGCCCTCA
cox10-SQ5F	GTGAAGAGTACAAGGCAG
cox10-R	TGTTATACAACGACGCC

1. Long N, Zeng L, Qiao S, Li L, Zhong G. 2018. Aspergillus fumigatus Afssn3-Afssn8 Pair Reverse Regulates Azole Resistance by Conferring Extracellular Polysaccharide, Sphingolipid Pathway Intermediates, and Efflux Pumps to Biofilm. Antimicrob Agents Chemother 62.
2. Chen P, Liu M, Zeng Q, Zhang Z, Liu W, Sang H, Lu L. 2019. Uncovering New Mutations Conferring Azole Resistance in the Aspergillus fumigatus cyp51A Gene. Front Microbiol 10:3127.
